# A Strategy to Identify Dominant Point Mutant Modifiers of a Quantitative Trait

**DOI:** 10.1534/g3.114.010595

**Published:** 2014-04-17

**Authors:** William F. Dove, Alexandra Shedlovsky, Linda Clipson, James M. Amos-Landgraf, Richard B. Halberg, Kathleen J. Krentz, Frederick J. Boehm, Michael A. Newton, David J. Adams, Thomas M. Keane

**Affiliations:** * McArdle Laboratory for Cancer Research, Department of Oncology, University of Wisconsin–Madison, Madison, Wisconsin 53706; † Laboratory of Genetics, University of Wisconsin–Madison, Madison, Wisconsin 53706; ‡ Department of Statistics, University of Wisconsin–Madison, Madison, Wisconsin 53706; § Department of Biostatistics and Medical Informatics, University of Wisconsin–Madison, Madison, Wisconsin 53706; ** Wellcome Trust Sanger Institute, Hinxton, Cambridge, UK

**Keywords:** intestinal neoplasia, genetic modifiers, ENU mutagenesis, isogenicity

## Abstract

A central goal in the analysis of complex traits is to identify genes that modify a phenotype. Modifiers of a cancer phenotype may act either intrinsically or extrinsically on the salient cell lineage. Germline point mutagenesis by ethylnitrosourea can provide alleles for a gene of interest that include loss-, gain-, or alteration-of-function. Unlike strain polymorphisms, point mutations with heterozygous quantitative phenotypes are detectable in both essential and nonessential genes and are unlinked from other variants that might confound their identification and analysis. This report analyzes strategies seeking quantitative mutational modifiers of *Apc^Min^* in the mouse. To identify a quantitative modifier of a phenotype of interest, a cluster of test progeny is needed. The cluster size can be increased as necessary for statistical significance if the founder is a male whose sperm is cryopreserved. A second critical element in this identification is a mapping panel free of polymorphic modifiers of the phenotype, to enable low-resolution mapping followed by targeted resequencing to identify the causative mutation. Here, we describe the development of a panel of six “isogenic mapping partner lines” for C57BL/6J, carrying single-nucleotide markers introduced by mutagenesis. One such derivative, B6.SNVg, shown to be phenotypically neutral in combination with *Apc^Min^*, is an appropriate mapping partner to locate induced mutant modifiers of the *Apc^Min^* phenotype. The evolved strategy can complement four current major initiatives in the genetic analysis of complex systems: the Genome-wide Association Study; the Collaborative Cross; the Knockout Mouse Project; and The Cancer Genome Atlas.

Genetic analysis of a phenotype in a multicellular organism starts with the discovery of those genes that influence the process in the whole organism. Loss-of-function alleles provide the least ambiguous route to understand the role, direct or indirect, of the functional wild-type allele in the process of interest. Gain-of-function and neomorphic alleles can provide further functional information. The action of the gene in the whole animal is ascertained by studying animals that are mosaic or chimeric for mutant and wild-type tissue (Hotta and Benzer 1972). When a gene of interest has been identified at the molecular level, longitudinal cellular studies provide complementary information on the spatial and temporal patterns of that gene’s actions.

Cancer in a metazoan is itself a multicellular process. Dramatic progress is emerging from The Cancer Genome Atlas (Vogelstein *et al.* 2013) and the Cancer Epigenome Project (Beck *et al.* 2012) to document mutations and epigenetic changes common to the neoplastic lineages of each cancer histotype. However, biologists recognize that a cancer is more than its genome. Beyond the epithelial lineage of the carcinoma, stromal elements can extrinsically influence the biology of the tumor. Such elements include leukocytes (Nosho *et al.* 2011), mast cells (Khan *et al.* 2013), neutrophils (Sinnamon *et al.* 2008), macrophages and cancer-associated fibroblasts (Edin *et al.* 2012; Herrera *et al.* 2013), and endothelial cells and the microvasculature of the tumor (Yekkala and Baudino 2007; Pitroda *et al.* 2012). Genes acting on the neoplastic lineage, intrinsically or extrinsically, to affect the cancer phenotype can be discovered using modifier genetics (Dietrich *et al.* 1993; Gould and Dove 1996; Cormier *et al.* 1997). Genome-wide discovery programs for polymorphic modifiers of cancer risk are being carried out both in human populations (Gabriel *et al.* 2002; Carvajal-Carmona *et al.* 2011; Peters *et al.* 2012; Cai *et al.* 2013) and in rodent models (Ewart-Toland *et al.* 2003; Elahi *et al.* 2009; Kwong and Dove 2009; Crist *et al.* 2011; Liu *et al.* 2011; Smits *et al.* 2011; Quan *et al.* 2011; Eversley *et al.* 2012; Nnadi *et al.* 2012). However, identifying the causative elements underlying a polymorphic quantitative risk modifier is a Herculean task (Drinkwater and Gould 2012; *e.g.*, see Lewis and Tomlinson 2012).

An important consideration is that the inbred strains of mice or rats that provide the starting material for modifier screens must carry functional alleles in all vital genes. Thus, the informative loss-of-function alleles of vital genes are absent from a modifier screen that emerges from an inbred line.

These considerations have driven us to design a strategy for the genome-wide discovery of mutagen-induced alleles that quantitatively modify the tumor-inducing phenotype of the dominant *Multiple intestinal neoplasia* (*Min*) allele of the *Adenomatous polyposis coli* (*Apc*) gene of the mouse (Moser *et al.* 1990). The mutagen *N*-ethyl-*N*-nitrosourea (ENU) predominantly causes single point mutations in the rodent germline. This simplicity has enabled facile identification of a growing set of genes, identified as ENU-induced mutant alleles (Su *et al.* 1992; King *et al.* 1997; Garcia-Garcia *et al.* 2005; Van Boxtel *et al.* 2010). ENU mutagenesis produces a range of point mutations (Takahasi *et al.* 2007). The frequency of such mutations in the sperm of an efficiently mutagenized mouse or rat is in the order of 10^−3^ per locus. A “one-hit library” has a size expected to contain, on average, one mutant for each locus. Thus, a one-hit mutational library of first-generation offspring of a mutagenized male has a size in the order of 1000 founders. By contrast, transposon-mediated mutagenesis of the mouse germline is significantly less efficient and does not yield the range of alleles produced by ENU.

Mutations causing a qualitative phenotype in monogenic traits can be identified without first being mapped, using identity-by-descent in genome-wide sequencing data (Arnold *et al.* 2011; Hilton *et al.* 2011). For example, two-generation screens can be used to identify new alleles of known genes (Shedlovsky *et al.* 1993) or new loci (Kumar *et al.* 2011). By contrast, individual components of a highly complex trait do not show qualitative patterns of inheritance. To detect a mutant allele that quantitatively modifies a phenotype of interest, a cluster of progeny must be phenotyped. Extensive progeny testing becomes feasible if the kindred’s founder is a male whose germline is cryopreserved to permit extensive retesting. Thus, quantitative modifying alleles for these traits are identified by low-resolution mapping followed by targeted sequence analysis to identify the causative mutation. Such mapping requires crossing the candidate founder to a partner carrying distinct marker alleles distributed over the genome. However, outcrossing to another inbred strain may introduce polymorphic modifiers from the donor mapping strain that can obfuscate the quantitative phenotype of interest (Elahi *et al.* 2009; Crist *et al.* 2011; Liu *et al.* 2011; Eversley *et al.* 2012; Nnadi *et al.* 2012). Therefore, we have employed germline mutagenesis, followed by inbreeding, to create a set of “isogenic mapping partners” for the canonical C57BL/6J (B6) mouse strain. To begin, one of these lines, B6.SNVg, has been shown by progeny testing to be phenotypically neutral in respect to the *Apc^Min^* phenotype. Resequencing of this line has established a set of induced single-nucleotide variant (SNV) markers covering the genome at sufficient density to make B6.SNVg a suitable partner for mapping a new ENU-induced dominant modifier of the *Apc^Min^* phenotype on the B6 background.

## Materials and Methods

### Mice

Mice were maintained under a protocol approved by the Animal Care and Use Committee of the University of Wisconsin School of Medicine and Public Health and in a facility in the McArdle Laboratory approved by the American Association of Laboratory Animal Care. Animals were housed in standard caging with free access to mouse chow and acidified water.

BTBR/Pas (BTBR) mice, initially obtained from J-L. Guénet at the Institut Pasteur in Paris, France, were inbred in our laboratory.

C57BL/6J-*Apc^Min^* (B6-*Apc^Min^*) mice were obtained from the McArdle colony. The strain was maintained by backcrossing every fifth generation to C57BL6/J (B6) mice from The Jackson Laboratory (Bar Harbor, ME). Progeny from B6-*Apc^Min/+^* mothers were fostered to ICR foster mothers to enhance their probability of survival.

Along with others, we have observed that dominant modifiers of the *Apc^Min^* phenotype arise spontaneously in the B6 stock (Baran *et al.* 2007). Therefore, we now maintain a closed colony, C57BL/6JD-*Apc^Min^* (D for Dove), and monitor it regularly for tumor multiplicity to prevent introgression of modifying alleles that can arise through genetic drift in the B6 stock. Through monitoring, the average tumor multiplicity in the closed colony is kept at approximately 100 ± 30 for mice at 90−120 d of age.

Lines B6.SNVb, B6.SNVc, B6.SNVe, B6.SNVf, B6.SNVg, and B6.SNVh were developed using ENU mutagenesis of B6 male founders, followed by at least 10 generations of inbreeding. They carry single nucleotide variant sets b, c, e, f, g, and h, respectively.

### ENU mutagenesis

BTBR males were mutagenized with 250 mg of ENU per kg body weight using the protocol previously described (Shedlovsky *et al.* 1986). Because B6 males are permanently sterilized at 250 mg of ENU per kg body, we mutagenized them with three weekly injections of 100 mg per kg body weight.

### Tumor scoring

Each intestinal tract was removed, opened longitudinally, and laid out on bibulous paper. The samples were fixed in 10% formalin overnight, then transferred to 70% ethanol for long-term storage. Tumor counts were obtained for the entire small intestine and colon using a Nikon SMZ-U dissecting microscope.

### Sequencing

Paired-end 75-bp reads were generated by end-sequencing DNA sheared to an average of 350 bp. Sequencing was performed on the Illumina GAIIx platform to an average depth of 5.7 times genome coverage (Supporting Information, Table S1). These data were mapped to the mm9/NCBIm37 assembly using BWA v0.5.9 (Li and Durbin 2009). Variants were called using Samtools (v0.1.17) mpileup/bcftools and then filtered selecting those variants for which the read depth was 3 or greater. Major changes in copy number or sequencing efficiency were culled by filtering out signals stronger than 2.5 times the sequencing coverage of the sample. Data are shown in File S1.

### Sequenom validation

In selecting candidates from line B6.SNVg for Sequenom validation, the goal was to find three candidates on each of the autosomes—near the centromere, middle, and telomere—fitting the following criteria:

Phred scale genotyping quality score of 99.
No significant repeats in flanking sequence.
Appearing in both B6.SNVg and B6.SNVh (known to be related), but in no other line.
A > T or T > A transversions were preferred, followed by A > G or T > C and G > A or C > T transitions. ENU mutagenesis enhances the proportion of AT to TA mutations, comprising 18.7% of the unique mutations in this study, in contrast to 9% of spontaneous mutations (14849300 of 164137670 B6.SNVs across 18 strains) (Keane *et al.* 2011).

Beyond the Phred scale quality rating of 99, the aforementioned criteria had to be relaxed in regions where mutations were sparse. Sequenom genotyping was performed as previously described (Gabriel *et al.* 2009). Chosen candidate variants in line B6.SNVg are validated in this communication; candidates in the other lines remain to be validated.

## Results

### Outcross design with clustered progeny testing

**Figure 1:**
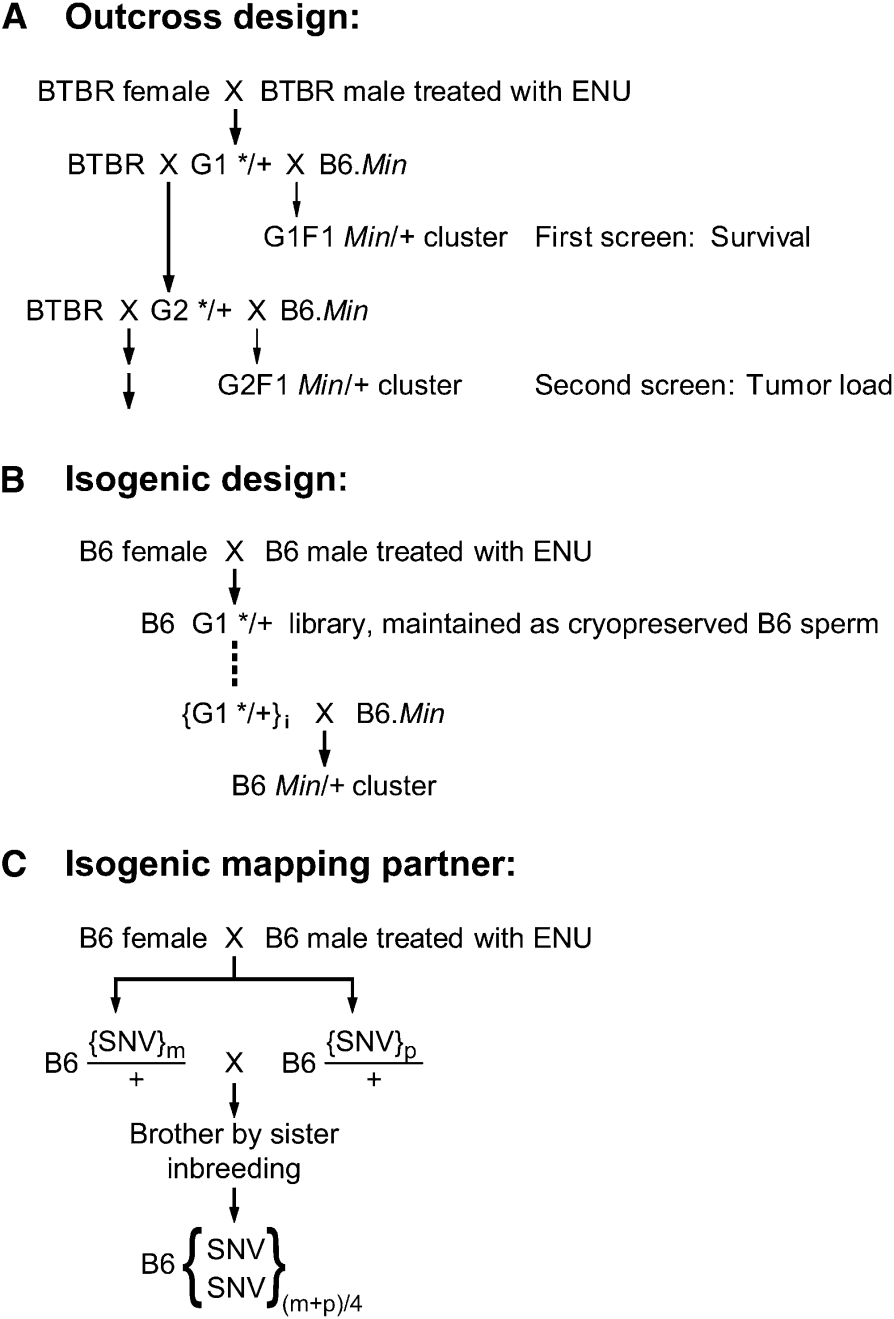
Breeding schemes to detect mutagen-induced dominant quantitative modifiers of the *Apc^Min^* phenotype. Three different designs are shown. The term “cluster” indicates that multiple progeny were screened to determine whether the kindred differed significantly from the mean. (A) Outcross modifier screen of BTBR G1 males based first upon survival and subsequently upon tumor multiplicity. (B) Isogenic modifier screen of B6 mutagenized males, coupled with the ability to save sperm from candidate animals. (C) Design for producing inbred B6.SNV lines from mutagenized B6 males. Note that inbreeding eliminates recessive lethal or detrimental mutations. The existing B6.SNV lines have been inbred for at least 10 generations. The residual heterozygosity expected at F10 is 14% (Green 1981).

Our initial strategy to discover ENU-induced alleles that quantitatively modify the *Apc^Min^* phenotype involved outcrossing a first-generation carrier of a mutagenized BTBR paternal genome to a B6-*Apc^Min/+^* tester. BTBR carries the same sensitive allele as B6 at the known modifier *Mom1* locus (Gould *et al.* 1996), leading us to choose this partner to B6-*Apc^Min^* in the outcross design. BTBR males, mutagenized as described in the section *Materials and Methods*, were bred to normal BTBR females to produce first-generation (G1) males that were potential carriers of *Apc^Min^* modifiers. These were first screened for such modifiers based upon the life span of their F1(B6-*Apc^Min/+^* X G1) offspring. Figure 1A shows the breeding designs used.

**Figure 2:**
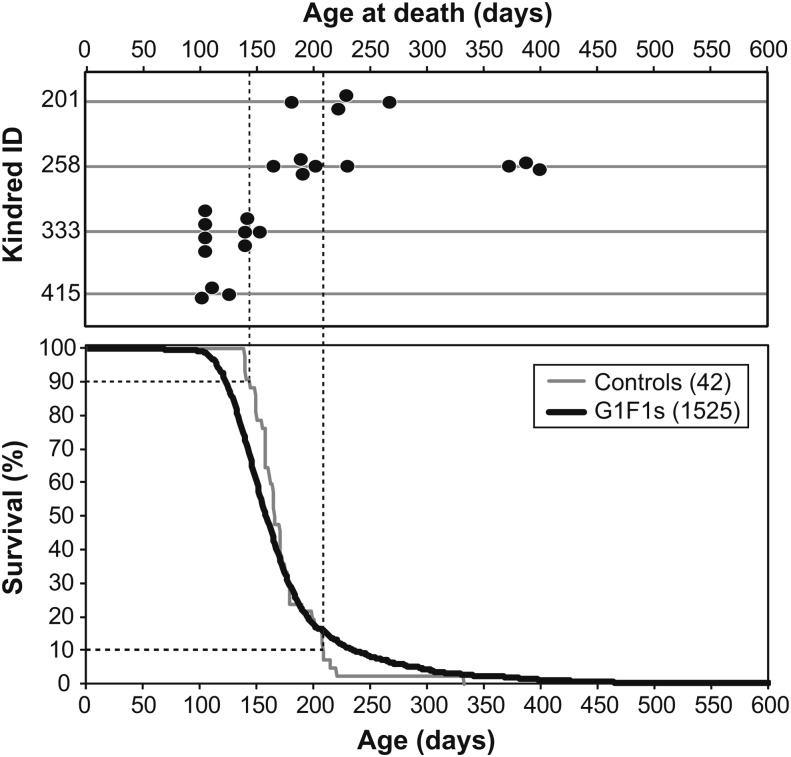
Candidate kindreds of dominant modifiers of *Apc^Min^* identified by survival but not successfully mapped. The lower panel shows the survival curve of F1(B6-*Apc^Min^* X BTBR) *Apc^Min^*^/+^ controls (gray line) and of *Apc^Min^*^/+^ progeny from mutagenized BTBR outcrossed to B6-*Apc^Min/+^* females (G1F1s, black line). The upper panel show the survival times of *Apc^Min^*^/+^ progeny from four different mutagenized BTBR G1 males (each the founder of a kindred). The four kindreds shown were chosen from 172 test kindreds that generated clusters of *Apc^Min/+^* progeny with extreme short or long lifespans.

The presenting phenotype in this initial foray was that of survival. Of 172 kindreds sired by G1 males, our attention was drawn to four kindreds carrying modifiers of the *Apc^Min^* survival phenotype: two putative dominant suppressors (258 and 201) and two putative dominant enhancers (333 and 415). Each of these kindreds was identified for further study by observing more than one animal with an extreme lifespan – long for the putative dominant suppressors and short for the putative dominant enhancers. Figure 2 displays the survival of control F1 (B6-*Apc^Min/+^* X BTBR) animals compared with F1s from all mutagenized males, as well as the survival of progeny within the aforementioned four kindreds.

**Figure 3:**
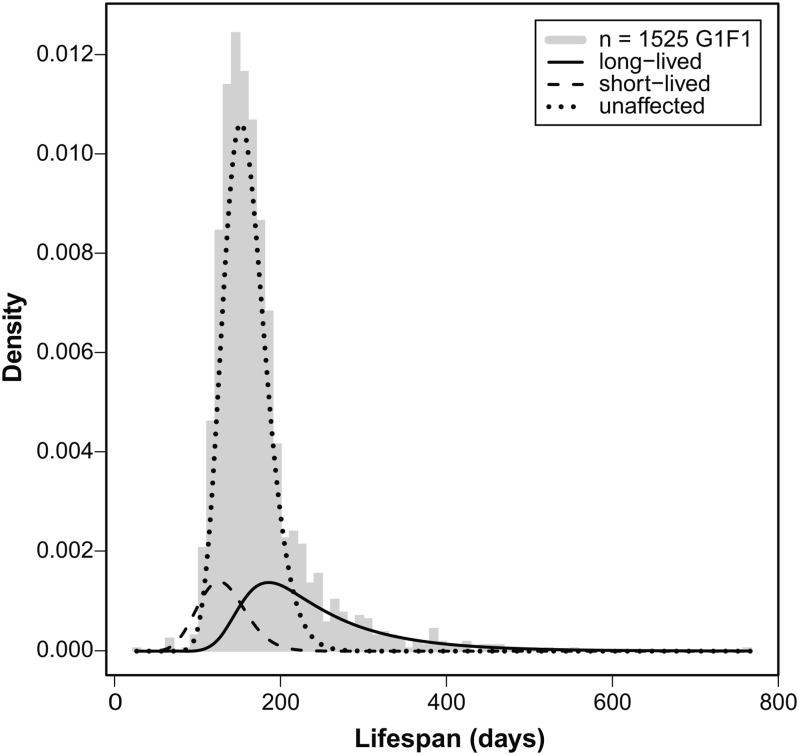
A statistical analysis of the differences in lifespan considers the distribution of mutagenized G1F1 Min animals as a mixture of three components (unaffected, long-lived, short-lived), and uses the maximum likelihood method to estimate both the rate at which ENU induces a phenotypic modifier and the distribution of multiplicative modifier effects (File S2). From this calculation, we estimated that 39% of the mutagenized kindreds are free of dominant modifier alleles, 41% carry dominant suppressor alleles that lead to reduced tumor multiplicity and longer lifespan, and 20% carry dominant enhancer alleles that have the opposite phenotype. A high proportion (61%) of mutagenized kindreds appear to carry modifiers of *Apc^Min^*, but most are of small effect. Equation (2) in File S2 gives the formula to estimate the enrichment in large modifier effects that can be achieved by selecting kindreds having multiple animals with an extreme survival phenotype.

Differences in the lifespan distribution between unaffected F1 animals and potentially affected G1F1 animals reflect both the rate and quantitative effect of ENU-induced modifiers. A statistical analysis of these differences considers the distribution of mutagenized G1F1 Min animals as a mixture of three components (unaffected, enhanced, suppressed), and uses the maximum likelihood method to estimate both the rate at which ENU induces a phenotypic modifier and the distribution of multiplicative modifier effects (Figure 3 and File S2). From this calculation, we estimate that 39% of the mutagenized kindreds are free of dominant modifier alleles, 41% carry dominant suppressor alleles that lead to reduced tumor multiplicity and longer lifespan, and 20% carry dominant enhancer alleles that have the opposite phenotype. Importantly, though a high proportion (61%) of mutagenized kindreds appears to carry modifiers of *Apc^Min^*, it is evident that most are of small effect^5^ (Kwong and Dove 2009). Equation (2) in File S2 gives a formula for the enrichment in large modifier effects that can be achieved by selecting kindreds having multiple animals with extreme phenotype, as was done with kindreds 201, 258, 333, and 415 (Figure S3 and Figure S4).

In practice, the quantitative character of this screen for dominant modifiers of the *Apc^Min^* phenotype was first analyzed on the basis of a simplifying assumption: that the time of survival of a *Apc^Min/+^* animal was correlated with its tumor multiplicity. The four candidate kindreds, first identified by survival, were further analyzed by tumor multiplicity of test progeny. The requirement for extensive progeny testing on the basis of both survival and tumor multiplicity led to an elapsed decision time that exceeded the lifespan of the founding first-generation mice. To implement this strategy, the germlines of these founders need to be maintained through sperm cryopreservation. However, recovery of BTBR/Pas from cryopreserved sperm remains elusive, while success with B6 sperm has only recently been significantly improved (Takeo and Nakagata 2011) and confirmed in our group by K.J.K.

### Isogenic design

Our attempts to map the presumptive dominant enhancer in kindred 333 and suppressor in kindred 258 were unsuccessful owing to modifying alleles polymorphic between BTBR/Pas and B6, such as *Mom7* (Kwong *et al.* 2007). Indeed, 18 such polymorphic modifier loci of the *Apc^Min^* tumor multiplicity phenotype have been reported to date (Eversley *et al.* 2012; Nnadi *et al.* 2012). The requirement for quantitative precision in this genetic strategy led us to move beyond the initial, informative outcross design and to develop an “isogenic design” (Figure 1B). This method requires a set of “isogenic mapping partners” for B6 that carry markers throughout the genome. To develop these partners we mutagenized several B6 males as described in the *Materials and Methods* and bred each to normal B6 females (Figure 1C). Progeny of these crosses were randomly selected to give seven brother by sister pairs to intercross for at least 10 generations. One line (B6.SNVd) failed to achieve 10 generations of inbreeding, presumably owing to homozygosity for a recessive lethal mutation. The other six lines achieved 10 or more generations of inbreeding without evident homozygosity for a recessive detrimental or lethal mutation. These B6.SNV lines, each with a set of SNVs came from separate lineages with the exception of two lines (B6.SNVg and h) that had a common first-generation parent.

### Progeny test of the B6.SNVg line for dominant modification of the *Apc^Min^* phenotype

**Figure 4:**
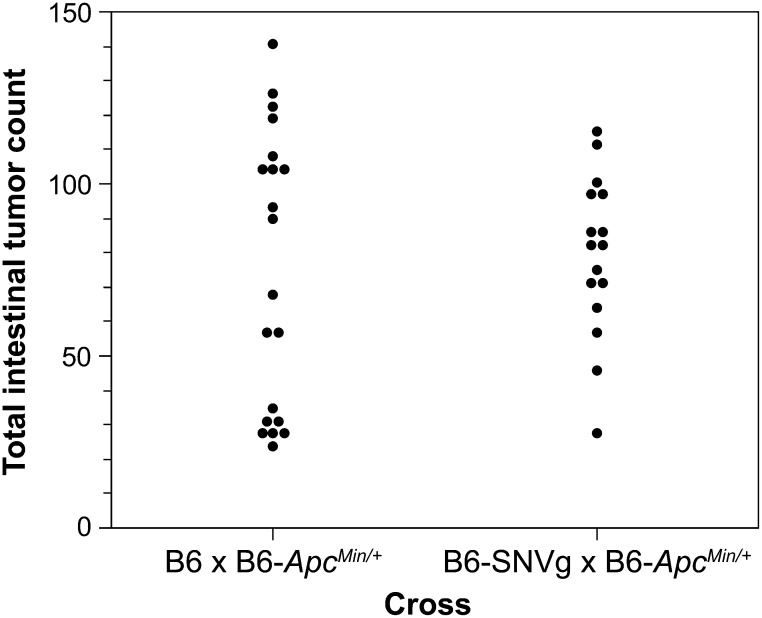
Progeny testing indicates that the B6.SNVg line carries no dominant modifiers of the *Apc^Min/+^* phenotype. B6-*Apc^Min/+^* males were each mated both to B6 females and B6.SNVg females. Tumors counts of *Apc^Min^*^/+^ progeny averaged 75 ± 40 for B6 x B6-*Apc^Min/+^* and 79 ± 23 for B6.SNVg × B6-*Apc^Min/+^* (*P* = 0.89, two-sided Wilcoxon rank sum test). The analysis has been limited to comparing two sets of progeny paired by B6-*Apc^Min/+^* sires in common, because the tumor multiplicities of our B6-*Apc^Min/+^* colony varied significantly over time until the closed colony C57BL/6JD was established.

The B6.SNVg line was tested for dominant modifiers of the *Apc^Min^* phenotype in crosses to B6-*Apc^Min^*. Females of the B6.SNVg line were bred to B6-*Apc^Min^* animals from the McArdle Laboratory stock. (B6.SNVg X B6-*Apc^Min^*) and *Apc^Min^*^/+^ progeny were killed at 90−100 d of age for tumor scoring. In parallel, tumor counts were established from (B6 X B6-*Apc^Min/+^*)F1 *Apc^Min^*^/+^ control progeny, sired by the same males (Figure 4). No significant difference was observed between the two groups (*P* = 0.89 by two-sided Wilcoxon Rank Sum test).

### Estimation of the size of the progeny test needed to detect a carrier of a mutant modifier of Min

**Figure 5:**
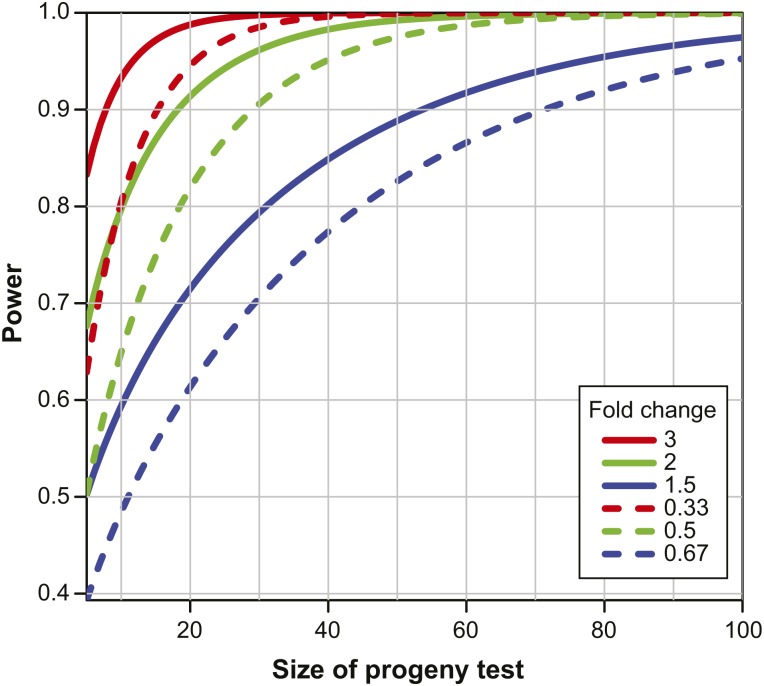
The number of progeny is estimated needed to test fully a mutagenized paternal gamete carried in a Generation 1 founder of a kindred. The null hypothesis tested is that the kindred carries no ENU-induced modifier. The tumor multiplicity data are assumed to follow a negative binomial distribution around a mean of 99.8 tumors and an overdispersion shape parameter of 9.8, as estimated from recent data from our closed colony, C57BL/6JD.*Apc^Min^*. If a modifier is carried by a kindred in heterozygous form, it is assumed to segregate and to affect only the tumor multiplicity and its variance, but not the shape parameter. The number of progeny is shown on the x-axis required to achieve sufficient power (on the y-axis) to detect at a 5% significance level a fold-effect given by the lines. The power calculations are based on the approximation to a normal distribution of the average tumor multiplicities within the test kindred.

The number of progeny needed to detect a Generation 1 carrier of an ENU-induced modifier at power 0.9 was estimated from the standard, overdistributed tumor multiplicity phenotype of B6-*Apc*^Min/+^ (File S2). In the first iteration of this assessment, we have used tumor multiplicity alone to score the phenotype. In a second iteration, we have first preselected kindreds that are more likely to contain modifiers on the basis of shortened or lengthened survival, as summarized above. In each case, we have used a negative binomial model similar to that described more fully in the legend to Figure 5. We ask that a reported list of kindreds, controlled at 5% false discovery rate, be nonempty with at least 95% probability. The dimensions of this progeny test at power 0.9 for specific magnitudes of dominant effect of an ENU-induced modifier are shown in Figure S1.

In the absence of preselection, for example on the basis of survival times, a prohibitive number of kindreds must be screened to the following depths. As shown in Figure S1A, to be 95% confident of discovering at least one allele with a twofold enhancing effect, 350 kindreds must be screened. For such a dominant suppressor allele, 500 kindreds would be needed.

These estimates indicate that a realistic screen for dominant enhancer and suppressor alleles of the overdistributed Min phenotype must involve either preselection of kindreds of interest or sharpening the *Apc^Min^* phenotype (Newton and Hastie 2006). The estimated distribution of the frequencies and fold effect of dominant enhancer and suppressor alleles in the population of Generation 1 founders (Figure 3) and the necessary sizes of test progeny clusters (Figure 5) lead in this case to the estimates summarized in Figure S1B. For example, to detect at least one dominant enhancer allele with a twofold effect, approximately 13 short-lived kindreds must be tested, each with 20 *Apc^Min/+^* progeny. The corresponding estimate to detect at least one dominant suppressor allele is 14 long-lived kindreds, each tested with 30 *Apc^Min/+^* progeny.

### Sequencing of the B6.SNV lines

Genomes of six of the B6.SNV lines were sequenced as described in *Materials and Methods*. We identified across all 6 B6.SNV lines a total of 22,911 high confidence candidate SNV positions from the sequencing data by comparison to the B6 (mm9/NCBIm37) reference genome (Table S1).

### A B6.SNVg mapping panel of confirmed SNV sites

Because progeny tests indicated that B6.SNVg does not dominantly affect the tumor multiplicity phenotype of B6-*Apc^Min^* (Figure 4), we have developed a mapping panel of SNV sites in this line. Thus, it can serve as the isogenic mapping partner to locate new ENU-induced dominant modifiers of the *Apc^Min^* phenotype.

A total of 180 SNV candidate marker sites were chosen for confirmation by Sequenom genotyping as described in the *Materials and Methods* with 148 entering the final assay. Validation was carried out on DNA samples from 31 C57BL/6JD, six B6.SNVg, one B6.SNVh, and 41 (C57BL/6JD x B6.SNVg)F1 mice. The results are displayed in File S2. Of the 148 candidate sites tested, 8 failed to give Sequenom results, so data are shown for 140 candidates. For 5 of these 140, the results may indicate either divergence between our C57BL/6JD and the canonical B6 used in sequencing NCBIm37, or else errors in that sequence. One candidate does not vary between B6.SNVg and C57BL/6JD, probably reflecting an error in the B6.SNVg sequence. Eleven of the candidates show evidence for residual heterozygosity in the B6.SNVg line, which is consistent with the expectation of 11% for 12 generations of inbreeding (Green 1981). This heterozygosity is not obligatory; the set of progeny includes B6.SNV/B6.SNV homozygotes at each of these eleven sites. Thus, 123 sites were validated as being ENU-derived and homozygous in B6.SNVg.

### The use of B6.SNVg as a mapping partner

**Figure 6:**
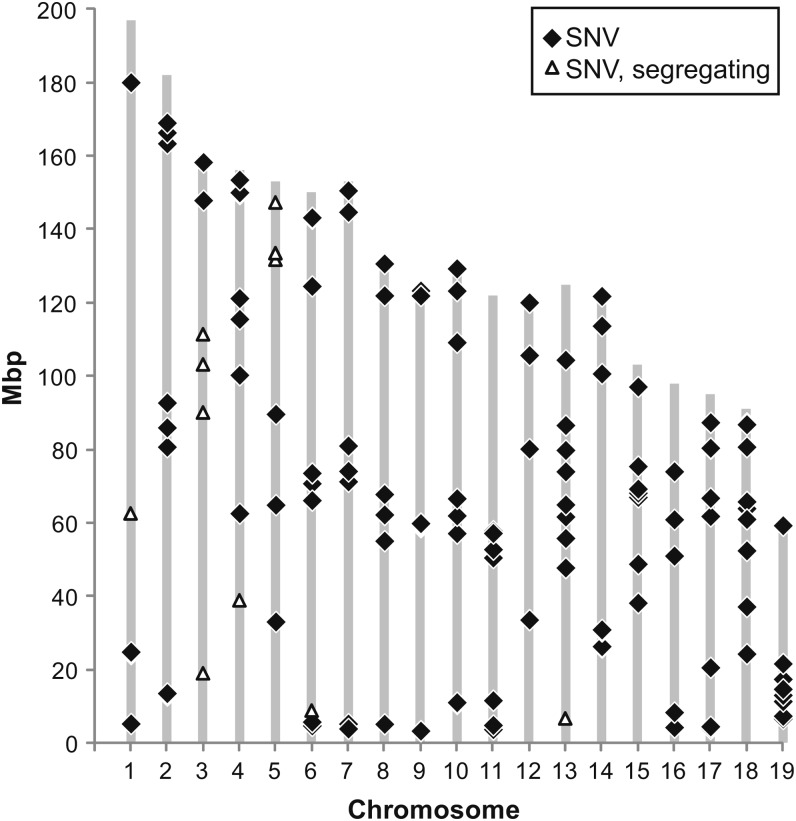
Location of point mutations in B6.SNVg that were validated by Sequenom testing. A total of 180 SNV candidate sites were chosen for confirmation by Sequenom genotyping, with 140 giving results in the final Sequenom assay. Shown are the 123 sites that are homozygous in B6.SNVg and the 11 sites showing residual heterozygosity. Validation was carried out on DNA samples from C57BL/6JD, B6.SNVg, B6.SNVh, and (C57BL/6JD x B6.SNVg)F1 mice (see File S4). The map displays the set of validated SNV sites that can serve the purpose of mapping to low resolution a newly induced dominant modifier allele by crosses of the B6 carrier of the new modifier to B6.SNVg.

Figure 6 presents the set of B6.SNVg sites that can serve the purpose of mapping a newly induced dominant modifier allele on B6. The meiotic maps of mammalian genomes show strong positive crossover interference (Broman *et al.* 2002). Thus, one expects that this mapping panel will permit a new mutation to be mapped to a defined region of the B6 genome. Targeted resequencing of this region in DNA from carriers of the newly induced modifier is then expected to yield one, or at most a small number, of candidate single base pair mutations; these can be confirmed by allelic substitution by the CRISP/Cas9 process (Wang *et al.* 2013).

### The frequency and pattern of ENU-induced mutations

Table S1 summarizes the resequencing analysis of the six B6.SNV lines. The frequency of presumptive mutations per base pair reported from the canonical sequence of B6 (NCBIm37/mm9) was 1.3 × 10^4^ in 6 × 2 × 10^9^ base pairs covered = 1.1 × 10^−6^ per base pair. This frequency is consistent with those previously reported (Michaud *et al.* 2005; Takahasi *et al.* 2007). Table 1 reports the numbers of line-specific mutations on each chromosome; Table 2 summarizes the spectrum of the unique mutations. Transitions from AT to GC and GC to AT ranked 1st and 2nd, whereas transversions between AT and TA ranked a close 3rd. This spectrum is similar to those reported in the study of Takahasi *et al.* (2007) and in the review by Barbaric *et al.* (2007).

**Table 1:** Distribution of line-specific candidate variants by chromosome and line

Chr	No. Candidate Variants						
	Only B6.SNVb	Only B6.SNVc	Only B6.SNVe	Only B6.SNVf	Only B6.SNVg	Only B6.SNVh	Only B6.SNVg & B6.SNVh
1	300	300	89	108	52	81	9
2	206	232	51	53	121	78	19
3	244	198	144	56	47	263	54
4	348	215	254	174	36	105	21
5	95	246	110	104	82	218	46
6	175	108	54	49	96	56	49
7	187	57	127	57	46	236	102
8	179	84	78	46	81	138	92
9	132	63	108	33	106	42	12
10	114	216	115	62	79	127	73
11	159	71	76	34	75	37	5
12	149	121	119	43	34	122	10
13	120	185	57	40	31	104	70
14	114	103	158	26	36	221	23
15	92	69	117	59	29	67	15
16	163	57	42	35	43	160	98
17	138	180	85	97	47	113	54
18	92	111	39	48	61	136	7
19	54	89	75	6	36	51	11
X	185	227	153	68	87	165	22
Total	3246	2932	2051	1198	1225	2520	792

In addition to the 13,172 candidate variants each found only in a single line, 792 other candidate variants are each found in lines B6.SNVg and B6.SNVh but in no other lines. B6.SNVg and B6.SNVh share founders, but the other lines are independent. Chr, chromosome.

**Table 2:** Mutational pathway of the 13,172 line-specific candidate variants

			Line-Specific Candidate Variants	
Mutation type	Ref	Alt	N	Percent
Transition	A	G	2142	16
	T	C	2497	19
	C	T	1540	12
	G	A	1435	11
Transversion	A	C	516	4
	T	G	598	5
	C	A	683	5
	G	T	649	5
	A	T	1114	8
	T	A	1352	10
	C	G	332	3
	G	C	314	2

### The distribution across the genome of the ENU-induced alleles in the SNV lines

Are regions of the genome of a SNV line devoid of markers, owing to homozygous lethality of mutations? The complete set of line-specific SNVs for the full set of B6.SNV lines is displayed in Figure S2. Full coverage of the genome, with some heterozygosity, is observed in three of the six lines—B6.SNVb, B6.SNVe, and B6.SNVg. At least one gap of over 30 Mpb is observed in each of the other lines. B6.SNVc is devoid of markers in two regions: Chr 7 at 100−144 Mbp; and Chr 11: 28−58 Mbp. B6.SNVf (whose resequencing coverage was only 1.7-fold) is devoid of markers in seven regions: Chr 5 at 43−77 Mbp; Chr 6 at 68−99 Mbp, Chr 11 at 89−121 Mbp, Chr. 12 at 26−67 Mbp; Chr 14 at 60−89 Mbp, Chr 16 at 43−77 Mbp; and Chr 1 at: 22−54 Mbp. B6.SNVh is devoid of markers on Chr 15 at 22−52 Mbp. Thus, in general the densely mutagenized genomes of the six B6.SNV lines support homozygosity, without loss of markers from particular regions.

## Discussion

We have developed a general genome-wide strategy to identify ENU-induced mutations that dominantly modify the *Apc^Min^* phenotype. In important ways this strategy complements others that are being actively pursued for the analysis of complex traits in mammalian biology such as cancer. The Genome-Wide Association Studies (GWAS, http://gwas.nih.gov/) and the Collaborative Cross (CC, http://csbio.unc.edu/CCstatus/index.py) reveal germline haplotypes that contribute to disease risk in the human and mouse population, respectively. By contrast, an allelic series of ENU-induced point mutations (see Table 2 in Takahasi *et al.* 2007) enables functional analysis of each gene affecting the trait of interest. Although loss-of-function alleles are particularly informative for cause-effect analysis, these alleles cannot be present among vital genes in the inbred mouse strains that generate the CC. The cancer genome and epigenome are addressed by The Cancer Genome Atlas (TCGA, http://cancergenome.nih.gov/) and the Cancer Epigenome Project (Beck *et al.* 2012). These projects identify mutations and other somatically heritable changes that act directly within the cancer lineage but not in the stroma. By contrast, modifying alleles can act in the tumor lineage, the stroma, or the host at large.

The detection of a subtle phenotype caused by a mutation in a single gene of a complex system is enhanced by amplifying the number of progeny displaying the mutant phenotype. Here, we describe a statistical approach to detect kindreds carrying dominant alleles that modify the tumor multiplicity phenotype. We have modeled briefly the null and modified distribution of the average tumor count within each kindred and calibrated this statistic, accounting for background phenotypic variation and modifier segregation. The power to detect a mutant signal is a function of the number of test progeny. Thus, the ability to recover a B6 line from cryopreserved sperm, to amplify a kindred under isogenic conditions, is a crucial element in implementing this quantitative modifier screen.

The advent of cost-effective sequencing of mammalian genomes led to the association (by identity-by-descent) of mutant phenotypes with particular segregating ENU-induced mutations. However, for genetically highly complex traits, it is likely to be necessary to delimit the genomic region carrying the new mutation of interest for targeted resequencing. The known point mutations preferentially caused by ENU simplify both the molecular identification of the gene of interest and the analysis of its mechanism of action. However, the distribution of distances found between adjacent candidate SNV mutations provides a measure of caution against the assumption that a single mutation is responsible for the mutant phenotype. Indeed, SNP sites in the human genome show evidence for nonrandom clustering (Amos 2010). We observe that the median distance (50th percentile) between in the SNV sites is 377,740 bp and the interquartile range is 48,852–1,115,595 (25th to 75th percentile) (Table S2). Thus, it is likely that a new modifier allele can be traced to a single mutation, but it will ultimately be necessary to validate the candidate by methods such as genome editing (Wang *et al.* 2013).

The evolved strategy discovers modifying genes on the basis of the heterozygous phenotype of an ENU-induced mutation. In what ways does this restriction limit the genome-wide coverage to discover the full set of genes that play a causative role in the biological process of interest? Application of mutagen-induced dominant modifier genetics broadens the genetic analysis by discovering loss-of-function alleles of vital genes that must be absent from inbred populations. Although such an allele can be maintained in heterozygous form, will the heterozygote have a phenotype? Alleles at quantitative trait loci commonly show additive inheritance. Many genes that modify a tumor pathway are haploinsufficient as judged by the quantitative phenotype of heterozygotes for knockout alleles. Examples include but are not limited to *Mmp7* (Wilson *et al.* 1997), T-cell factor 4 (Angus-Hill *et al.* 2011), Kruppel-like factors 4 (Ghaleb *et al.* 2007) and 5 (McConnell *et al.* 2009), *Park2* (Poulogiannis *et al.* 2010), E3 ubiquitin ligase Fbw7 (Sancho *et al.* 2010), SGO1 (Yamada *et al.* 2012), cMyc (Yekkala and Baudino 2007), and *SMAD4* (Szigeti *et al.* 2012). The quantitative nature of tumor suppression has been reviewed by Largaespada (2001) and Payne and Kemp (2005). Berger *et al.* (2011) have generalized the dosage sensitivity hypothesis for tumor suppressor genes. Loss-of-function alleles are maximally informative in the analysis of chains of causation. As discussed previously, for such alleles, the evolved strategy is biased toward cases of dominant alleles involving haploinsufficiency. This bias may favor the identification of dosage-sensitive targets of drug action.

B6.SNVg has been shown to be a phenotypically neutral isogenic partner for mapping new modifiers of B6-*Apc^Min^*. All six B6.SNV lines are available from the McArdle Laboratory for Cancer Research at the University of Wisconsin to be developed for research purposes as candidate mapping partners for the modifier genetics of the quantitative phenotype displayed by other B6 strains carrying a dominant element such as a transgene that affects a biological pathway of interest. The sequence changes detected by Illumina resequencing of these strains are shown in File S1. Only the calls in strain B6.SNVg were further tested by Sequenom analysis (File S3).

Other derivatives of B6 that have acquired new mutations by neutral drift or by strain admixture, such C57BL/6N, may also serve as isogenic mapping partners if shown to be phenotypically neutral (Simon *et al.* 2013).

We can anticipate significant future enhancements in the modifier genetics of complex traits in experimental mammals. On the phenotypic side, we can expect advances in the molecular and cellular dissection of tumor pathways. The complexity of cancer as a genetic trait surely includes the biological complexity of the disease. Subdividing the phenotype enhances the statistical power to detect a modifying mutation through its selective action on one component of such a complex system (see March *et al.* 2011; Green *et al.* 2011).This strategy has been demonstrated for circadian biology by Takahashi and his colleagues (Chen *et al.* 2012). On the genetic side, we can expect further improvements by a new generation of mapping partners: using the efficient one-step allelic substitution process (Wang *et al.* 2013), a geneticist can engineer an isogenic mapping partner of an inbred strain by introducing a planned set of SNV mapping sites genome-wide, designed to be functionally neutral.^6^

A genome-wide genetic screen for mutagen-induced quantitative modifiers of the complex *Apc^Min^* tumor phenotype can expand in important directions our molecular and cellular understanding of the complexities of intestinal neoplasia. This report illustrates the obstacles that must be overcome to achieve this end, and documents the creation of isogenic mapping partners for the canonical mouse strain C57BL/6J by which such sensitive genetic screens can be developed. This strategy and its *sequelae* can complement other active programs for the analysis of complex biological processes.

## Supplementary Material

Supporting Information

## Footnotes

5 “When one tugs at a single thing in nature, he finds it attached to the rest of the world.”—John Muir

6 “Isogenicity is like virtue. It is usually desired but seldom achieved.”—H. J. Muller

## References

[bib1] Amos W., 2010 Even small SNP clusters are non-randomly distributed: is this evidence of mutational non-independence? Proc. Biol. Sci. 277: 1443–1449 20071383 10.1098/rspb.2009.1757 PMC2871933

[bib2] Angus-Hill M. L. Elbert K. M. Hidalgo J. Capecchi M. R., 2011 T-cell factor 4 functions as a tumor suppressor whose disruption modulates colon cell proliferation and tumorigenesis. Proc. Natl. Acad. Sci. USA 108: 4914–4919 21383188 10.1073/pnas.1102300108 PMC3064334

[bib3] Arnold C. N. Xia Y. Lin P. Ross C. Schwander M., 2011 Rapid identification of a disease allele in mouse through whole genome sequencing and bulk segregation analysis. Genetics 187: 633–641 21196518 10.1534/genetics.110.124586 PMC3063661

[bib4] Baran A. A. Silverman K. A. Zeskand J. Koratkar R. Palmer A., 2007 The modifier of Min 2 (Mom2) locus: embryonic lethality of a mutation in the Atp5a1 gene suggests a novel mechanism of polyp suppression. Genome Res. 17: 566–576 17387143 10.1101/gr.6089707 PMC1855180

[bib5] Barbaric I. Wells S. Russ A. Dear T. N., 2007 Spectrum of ENU-induced mutations in phenotype-driven and gene-driven screens in the mouse. Environ. Mol. Mutagen. 48: 124–142 17295309 10.1002/em.20286

[bib6] Beck S. Bernstein B. E. Campbell R. M. Costello J. F. Dhanak D., 2012 A blueprint for an international cancer epigenome consortium. A report from the AACR Cancer Epigenome Task Force. Cancer Res. 72: 6319–6324 23188507 10.1158/0008-5472.CAN-12-3658

[bib7] Berger A. H. Knudson A. G. Pandolfi P. P., 2011 A continuum model for tumour suppression. Nature 476: 163–169 21833082 10.1038/nature10275 PMC3206311

[bib8] Broman K. W. Rowe L. B. Churchill G. A. Paigen K., 2002 Crossover interference in the mouse. Genetics 160: 1123–1131 11901128 10.1093/genetics/160.3.1123 PMC1462020

[bib9] Cai M. Dai H. Qiu Y. Zhao Y. Zhang R., 2013 SNP set association analysis for genome-wide association studies. PLoS ONE 8: e62495 23658731 10.1371/journal.pone.0062495 PMC3643925

[bib10] Carvajal-Carmona L. G. Cazier J. B. Jones A. M. Howarth K. Broderick P., 2011 Fine-mapping of colorectal cancer susceptibility loci at 8q23.3, 16q22.1 and 19q13.11: refinement of association signals and use of in silico analysis to suggest functional variation and unexpected candidate target genes. Hum. Mol. Genet. 20: 2879–2888 21531788 10.1093/hmg/ddr190 PMC3118761

[bib11] Chen Z. Yoo S. H. Park Y. S. Kim K. H. Wei S., 2012 Identification of diverse modulators of central and peripheral circadian clocks by high-throughput chemical screening. Proc. Natl. Acad. Sci. USA 109: 101–106 22184224 10.1073/pnas.1118034108 PMC3252927

[bib12] Cormier R. T. Hong K. H. Halberg R. B. Hawkins T. L. Richardson P., 1997 Secretory phospholipase Pla2g2a confers resistance to intestinal tumorigenesis. Nat. Genet. 17: 88–91 9288104 10.1038/ng0997-88

[bib13] Crist R. C. Roth J. J. Lisanti M. P. Siracusa L. D. Buchberg A. M., 2011 Identification of Mom12 and Mom13, two novel modifier loci of Apc (Min) -mediated intestinal tumorigenesis. Cell Cycle 10: 1092–1099 21386660 10.4161/cc.10.7.15089 PMC3100885

[bib14] Dietrich W. F. Lander E. S. Smith J. S. Moser A. R. Gould K. A., 1993 Genetic identification of Mom-1, a major modifier locus affecting Min-induced intestinal neoplasia in the mouse. Cell 75: 631–639 8242739 10.1016/0092-8674(93)90484-8

[bib15] Drinkwater N. R. Gould M. N., 2012 The long path from QTL to gene. PLoS Genet. 8: e1002975 23049490 10.1371/journal.pgen.1002975 PMC3462162

[bib16] Edin S. Wikberg M. L. Dahlin A. M. Rutegard J. Oberg A., 2012 The distribution of macrophages with a M1 or M2 phenotype in relation to prognosis and the molecular characteristics of colorectal cancer. PLoS ONE 7: e47045 23077543 10.1371/journal.pone.0047045 PMC3471949

[bib17] Elahi E. Suraweera N. Volikos E. Haines J. Brown N., 2009 Five quantitative trait Loci control radiation-induced adenoma multiplicity in Mom 1R Apc Min/+ mice. PLoS ONE 4: e4388 19194513 10.1371/journal.pone.0004388 PMC2633613

[bib18] Eversley C. D. Yuying X. Pearsall R. S. Threadgill D. W., 2012 Mapping six new susceptibility to colon cancer (scc) Loci using a mouse interspecific backcross. G3 (Bethesda) 2: 1577–1584 23275880 10.1534/g3.112.002253 PMC3516479

[bib19] Ewart-Toland A. Briassouli P. de Koning J. P. Mao J. H. Yuan J., 2003 Identification of Stk6/STK15 as a candidate low-penetrance tumor-susceptibility gene in mouse and human. Nat. Genet. 34: 403–412 12881723 10.1038/ng1220

[bib21] Gabriel S. Ziaugra L. Tabbaa D., 2009 SNP genotyping using the Sequenom MassARRAY iPLEX platform. Curr. Protoc. Hum. Genet. 60: 2.12.1–2.12.16 10.1002/0471142905.hg0212s60 19170031

[bib22] Gabriel S. B. Schaffner S. F. Nguyen H. Moore J. M. Roy J., 2002 The structure of haplotype blocks in the human genome. Science 296: 2225–2229 12029063 10.1126/science.1069424

[bib23] Garcia-Garcia M. J. Eggenschwiler J. T. Caspary T. Alcorn H. L. Wyler M. R., 2005 Analysis of mouse embryonic patterning and morphogenesis by forward genetics. Proc. Natl. Acad. Sci. USA 102: 5913–5919 15755804 10.1073/pnas.0501071102 PMC1087930

[bib24] Ghaleb A. M. McConnell B. B. Nandan M. O. Katz J. P. Kaestner K. H., 2007 Haploinsufficiency of Kruppel-like factor 4 promotes adenomatous polyposis coli dependent intestinal tumorigenesis. Cancer Res. 67: 7147–7154 17671182 10.1158/0008-5472.CAN-07-1302 PMC2373271

[bib25] Gould K. A. Dove W. F., 1996 Action of Min and Mom1 on neoplasia in ectopic intestinal grafts. Cell Growth Differ. 7: 1361–1368 8891340

[bib26] Gould K. A. Luongo C. Moser A. R. McNeley M. K. Borenstein N., 1996 Genetic evaluation of candidate genes for the Mom1 modifier of intestinal neoplasia in mice. Genetics 144: 1777–1785 8978063 10.1093/genetics/144.4.1777 PMC1207727

[bib27] Green E., 1981 Genetics and Probability in Animal Breeding Experiments. Oxford University Press, New York

[bib28] Green R. A. Kao H. L. Audhya A. Arur S. Mayers J. R., 2011 A high-resolution *C. elegans* essential gene network based on phenotypic profiling of a complex tissue. Cell 145: 470–482 21529718 10.1016/j.cell.2011.03.037 PMC3086541

[bib29] Herrera M. Herrera A. Dominguez G. Silva J. Garcia V., 2013 Cancer-associated fibroblast and M2 macrophage markers together predict outcome in colorectal cancer patients. Cancer Sci. 104: 437–444 23298232 10.1111/cas.12096 PMC7657228

[bib30] Hilton J. M. Lewis M. A. Grati M. Ingham N. Pearson S., 2011 Exome sequencing identifies a missense mutation in Isl1 associated with low penetrance otitis media in dearisch mice. Genome Biol. 12: R90 21936904 10.1186/gb-2011-12-9-r90 PMC3308053

[bib31] Hotta Y. Benzer S., 1972 Mapping of behaviour in *Drosophila* mosaics. Nature 240: 527–535 4568399 10.1038/240527a0

[bib32] Keane T. M. Goodstadt L. Danecek P. White M. A. Wong K., 2011 Mouse genomic variation and its effect on phenotypes and gene regulation. Nature 477: 289–294 21921910 10.1038/nature10413 PMC3276836

[bib33] Khan M. W. Keshavarzian A. Gounaris E. Melson J. E. Cheon E., 2013 PI3K/AKT signaling is essential for communication between tissue infiltrating mast cells, macrophages, and epithelial cells in colitis-induced cancer. Clin. Cancer Res. 19: 2342–2354 23487439 10.1158/1078-0432.CCR-12-2623 PMC3947836

[bib34] King D. P. Zhao Y. Sangoram A. M. Wilsbacher L. D. Tanaka M., 1997 Positional cloning of the mouse circadian clock gene. Cell 89: 641–653 9160755 10.1016/s0092-8674(00)80245-7 PMC3815553

[bib35] Kumar V. Kim K. Joseph C. Thomas L. C. Hong H., 2011 Second-generation high-throughput forward genetic screen in mice to isolate subtle behavioral mutants. Proc. Natl. Acad. Sci. USA 108: 15557–15564 21896739 10.1073/pnas.1107726108 PMC3176609

[bib36] Kwong L. N. Dove W. F., 2009 APC and its modifiers in colon cancer. Adv. Exp. Med. Biol. 656: 85–106 19928355 10.1007/978-1-4419-1145-2_8 PMC3754875

[bib37] Kwong L. N. Shedlovsky A. Biehl B. S. Clipson L. Pasch C. A., 2007 Identification of *Mom7*, a novel modifier of *Apc*^Min/+^ on mouse chromosome 18. Genetics 176: 1237–1244 17435219 10.1534/genetics.107.071217 PMC1894587

[bib38] Largaespada D. A., 2001 Haploinsufficiency for tumor suppression: the hazards of being single and living a long time. J. Exp. Med. 193: F15–F18 11181707 10.1084/jem.193.4.f15 PMC2195912

[bib39] Lewis A. Tomlinson I., 2012 Cancer. The utility of mouse models in post-GWAS research. Science 338: 1301–1302 23224545 10.1126/science.1231733

[bib40] Li H. Durbin R., 2009 Fast and accurate short read alignment with Burrows-Wheeler transform. Bioinformatics 25: 1754–1760 19451168 10.1093/bioinformatics/btp324 PMC2705234

[bib41] Liu P. Liu H. B. Lu Y. Wen W. Jia D., 2011 Genome-wide association and fine mapping of genetic loci predisposing to colon carcinogenesis in mice. Mol. Cancer Res. 10: 66–74 22127497 10.1158/1541-7786.MCR-10-0540

[bib42] March H. N. Rust A. G. Wright N. A. Ten Hoeve J. de Ridder J., 2011 Insertional mutagenesis identifies multiple networks of cooperating genes driving intestinal tumorigenesis. Nat. Genet. 43: 1202–1209 22057237 10.1038/ng.990 PMC3233530

[bib43] McConnell B. B. Bialkowska A. B. Nandan M. O. Ghaleb A. M. Gordon F. J., 2009 Haploinsufficiency of Kruppel-like factor 5 rescues the tumor-initiating effect of the Apc(Min) mutation in the intestine. Cancer Res. 69: 4125–4133 19435907 10.1158/0008-5472.CAN-08-4402 PMC2702486

[bib44] Michaud E. J. Culiat C. T. Klebig M. L. Barker P. E. Cain K. T., 2005 Efficient gene-driven germ-line point mutagenesis of C57BL/6J mice. BMC Genomics 6: 164 16300676 10.1186/1471-2164-6-164 PMC1325271

[bib45] Moser A. R. Pitot H. C. Dove W. F., 1990 A dominant mutation that predisposes to multiple intestinal neoplasia in the mouse. Science 247: 322–324 2296722 10.1126/science.2296722

[bib46] Newton M. A. Hastie D. I., 2006 Assessing Poisson variation of intestinal tumor multiplicity in Min mice carrying a Robertsonian translocation. Appl. Stat. 15: 123–138

[bib47] Nnadi S. C. Watson R. Innocent J. Gonye G. E. Buchberg A. M., 2012 Identification of five novel modifier loci of ApcMin harbored in theBXH14 recombinant inbred strain. Carcinogenesis 33: 1589–1597 22637734 10.1093/carcin/bgs185 PMC3499057

[bib48] Nosho K. Dranoff G. Fuchs C. Ogino S., 2011 Tumor-infiltrating CD45RO+-cell density, but not CD3+, CD8+, or FOXP3+-cell density, has a prognostic role in colorectal cancer, independent of molecular features. Lab. Invest. 91: 160A

[bib49] Payne S. R. Kemp C. J., 2005 Tumor suppressor genetics. Carcinogenesis 26: 2031–2045 16150895 10.1093/carcin/bgi223

[bib50] Peters U. Hutter C. M. Hsu L. Schumacher F. R. Conti D. V., 2012 Meta-analysis of new genome-wide association studies of colorectal cancer risk. Hum. Genet. 131: 217–234 21761138 10.1007/s00439-011-1055-0 PMC3257356

[bib51] Pitroda S. P. Zhou T. Sweis R. F. Filippo M. Labay E., 2012 Tumor endothelial inflammation predicts clinical outcome in diverse human cancers. PLoS ONE 7: e46104 23056240 10.1371/journal.pone.0046104 PMC3464251

[bib52] Poulogiannis G. McIntyre R. E. Dimitriadi M. Apps J. R. Wilson C. H., 2010 PARK2 deletions occur frequently in sporadic colorectal cancer and accelerate adenoma development in Apc mutant mice. Proc. Natl. Acad. Sci. USA 107: 15145–15150 20696900 10.1073/pnas.1009941107 PMC2930574

[bib53] Quan L. Stassen A. P. Ruivenkamp C. A. van Wezel T. Fijneman R. J., 2011 Most lung and colon cancer susceptibility genes are pair-wise linked in mice, humans and rats. PLoS ONE 6: e14727 21390212 10.1371/journal.pone.0014727 PMC3044722

[bib54] Sancho R. Jandke A. Davis H. Diefenbacher M. E. Tomlinson I., 2010 F-box and WD repeat domain-containing 7 regulates intestinal cell lineage commitment and is a haploinsufficient tumor suppressor. Gastroenterology 139: 929–941 20638938 10.1053/j.gastro.2010.05.078

[bib55] Shedlovsky A. Guenet J. L. Johnson L. L. Dove W. F., 1986 Induction of recessive lethal mutations in the T/t-H-2 region of the mouse genome by a point mutagen. Genet. Res. 47: 135–142 3710160 10.1017/s0016672300022977

[bib56] Shedlovsky A. McDonald J. D. Symula D. Dove W. F., 1993 Mouse models of human phenylketonuria. Genetics 134: 1205–1210 8375656 10.1093/genetics/134.4.1205 PMC1205587

[bib57] Simon M. M. Greenaway S. White J. K. Fuchs H. Gailus-Durner V., 2013 A comparative phenotypic and genomic analysis of C57BL/6J and C57BL/6N mouse strains. Genome Biol. 14: R82 23902802 10.1186/gb-2013-14-7-r82 PMC4053787

[bib58] Sinnamon M. J. Carter K. J. Fingleton B. Matrisian L. M., 2008 Matrix metalloproteinase-9 contributes to intestinal tumourigenesis in the adenomatous polyposis coli multiple intestinal neoplasia mouse. Int. J. Exp. Pathol. 89: 466–475 19134056 10.1111/j.1365-2613.2008.00621.x PMC2669608

[bib59] Smits B. M. Sharma D. Samuelson D. J. Woditschka S. Mau B., 2011 The non-protein coding breast cancer susceptibility locus Mcs5a acts in a non-mammary cell-autonomous fashion through the immune system and modulates T-cell homeostasis and functions. Breast Cancer Res. 13: R81 21846333 10.1186/bcr2933 PMC3236344

[bib60] Su L. K. Kinzler K. W. Vogelstein B. Preisinger A. C. Moser A. R., 1992 Multiple intestinal neoplasia caused by a mutation in the murine homolog of the APC gene. Science 256: 668–670 1350108 10.1126/science.1350108

[bib61] Szigeti R. Pangas S. A. Nagy-Szakal D. Dowd S. E. Shulman R. J., 2012 SMAD4 haploinsufficiency associates with augmented colonic inflammation in select humans and mice. Ann. Clin. Lab. Sci. 42: 401–408 23090737 PMC3875295

[bib62] Takahasi K. R. Sakuraba Y. Gondo Y., 2007 Mutational pattern and frequency of induced nucleotide changes in mouse ENU mutagenesis. BMC Mol. Biol. 8: 52 17584492 10.1186/1471-2199-8-52 PMC1914352

[bib63] Takeo T. Nakagata N., 2011 Reduced glutathione enhances fertility of frozen/thawed C57BL/6 mouse sperm after exposure to methyl-beta-cyclodextrin. Biol. Reprod. 85: 1066–1072 21778138 10.1095/biolreprod.111.092536

[bib64] van Boxtel R. Gould M. N. Cuppen E. Smits B. M., 2010 ENU mutagenesis to generate genetically modified rat models. Methods Mol. Biol. 597: 151–167 20013232 10.1007/978-1-60327-389-3_11

[bib65] Vogelstein B. Papadopoulos N. Velculescu V. E. Zhou S. Diaz L. A. Jr, 2013 Cancer genome landscapes. Science 339: 1546–1558 23539594 10.1126/science.1235122 PMC3749880

[bib66] Wang H. Yang H. Shivalila C. S. Dawlaty M. M. Cheng A. W., 2013 One-step generation of mice carrying mutations in multiple genes by CRISPR/Cas-mediated genome engineering. Cell 153: 910–918 23643243 10.1016/j.cell.2013.04.025 PMC3969854

[bib67] Wilson C. L. Heppner K. J. Labosky P. A. Hogan B. L. Matrisian L. M., 1997 Intestinal tumorigenesis is suppressed in mice lacking the metalloproteinase matrilysin. Proc. Natl. Acad. Sci. USA 94: 1402–1407 9037065 10.1073/pnas.94.4.1402 PMC19803

[bib68] Yamada H. Y. Yao Y. Wang X. Zhang Y. Huang Y., 2012 Haploinsufficiency of SGO1 results in deregulated centrosome dynamics, enhanced chromosomal instability and colon tumorigenesis. Cell Cycle 11: 479–488 22262168 10.4161/cc.11.3.18994 PMC3315092

[bib69] Yekkala K. Baudino T. A., 2007 Inhibition of intestinal polyposis with reduced angiogenesis in ApcMin/+ mice due to decreases in c-Myc expression. Mol. Cancer Res. 5: 1296–1303 18171987 10.1158/1541-7786.MCR-07-0232

