# Supplementary material for: A Strategy to Identify Dominant Point Mutant Modifiers of a Quantitative Trait
Source: G3 (Bethesda). 2014 Apr 17;4(6):1113–21. doi: 10.1534/g3.114.010595 (PMC4065254; doi:10.1534/g3.114.010595)
Supplement: Supporting Information [file supp_4_6_1113__index.html]

A Strategy To Identify Dominant Point Mutant Modifiers of a Quantitative Trait — A Strategy to Identify Dominant Point Mutant Modifiers of a Quantitative Trait — Supporting Information 

# A Strategy to Identify Dominant Point Mutant Modifiers of a Quantitative Trait

## Supporting Information for Dove *et al.*, 2014

**Files in this Data Supplement:**

- Supporting Information - Tables S1-S2, Figures S1-S4, and Files S1-S4 (PDF, 1 MB)
- Table S1 - Sequencing of the B6-SNV lines. (PDF, 111 KB)
- Table S2 - The spectrum of distances between 13172 adjacent line-specific candidate variants. (PDF, 112 KB)
- Figure S1 - Sample size requirements in a progeny test for various fold effects modifying the expected tumor count, both (A) without prescreening, assuming 1/100 gametes have a modifier with directional effect shown, and (B) with survival-based pre-screening such that ¼ of gametes have directional effect shown. (PDF, 268 KB)
- Figure S2 - The candidate SNV sites that are line-specific or specific to only lines B6.SNVg and B6.SNVh. (PDF, 864 KB)
- Figure S3 - *P*(*M* > *f*|SEL): how selecting on long-lived phenotype enriches for large fold effects. (PDF, 139 KB)
- Figure S4 - *P*(1/*M* > *f*|SEL): how selecting on short-lived phenotype enriches for small fold effects. (PDF, 137 KB)
- File S1 - Supporting Data - Illumina sequencing results for lines B6.SNVb, B6.SNVc, B6.SNVe, B6.SNVf, B6.SNVg and B6.SNVh. (.zip, 396 KB)
- File S2 - Supporting Methods - Modeling lifespans of mutagenized G1F1 animals. (.zip, 307 KB)
- File S3 - Supporting Methods - Mutagenesis sample-size planning. (.zip, 210 KB)
- File S4 - Supporting Data (.zip, 161 KB)
