# Supplementary material for: A Strategy to Identify Dominant Point Mutant Modifiers of a Quantitative Trait
Source: G3 (Bethesda). 2014 Apr 17;4(6):1113–21. doi: 10.1534/g3.114.010595 (PMC4065254; doi:10.1534/g3.114.010595)
Supplement: Supporting Information [file supp_g3.114.010595_010595SI.pdf]

G3 · Genes | Genomes | Genetics

A strategy to identify dominant mutant modifiers of a quantitative trait

William F. Dove[*,§,1], Alexandra Shedlovsky[*], Linda Clipson[*], James M. Amos-Landgraf[*,a], Richard B. Halberg[*,b],, Kathleen J. Krentz[*,c], Frederick J. Boehm[†], Michael A. Newton[†,‡], David J. Adams[**], Thomas M. Keane[**]

[*]McArdle Laboratory for Cancer Research, Department of Oncology, [§]Laboratory of Genetics, [†]Department of Statistics, [‡]Department of Biostatistics and Medical Informatics, University of Wisconsin – Madison, Madison, Wisconsin, USA.
[**]Wellcome Trust Sanger Institute, Hinxton, Cambridge, UK

[1]Corresponding author: 1400 University Avenue, Madison, Wisconsin 53706, USA. Email: dove@oncology.wisc.edu

DOI: 10.1534/g3.114.010595

Table S1 Sequencing of the B6-SNV lines.

Line	Raw (Gbp)	Mapped (Gbp)	Coverage (times)	N of all candidate SNVs[a]	N of line-specific candidate SNVs[b]
SNVb	27.40	23.83	7.94	9864	3246
SNVc	20.61	17.21	5.74	9028	2932
SNVe	14.64	12.24	4.08	7094	2051
SNVf	6.05	5.14	1.71	2925	1198
SNVg	28.38	24.25	8.08	6324	1225
SNVh	23.85	20.03	6.68	9559	2520

[a]There were 22911 sites overall: 13172 appeared in only 1 line, 3590 in 2, 2187 in 3, 2212 in 4, 1467 in 5, and 283 in all 6 lines.

[b]In addition, lines B6.SNVg and B6.SNVh, which are known to be related, share 792 candidate variants that are found in none of the other lines.

Table S2 The spectrum of distances between 13172 adjacent line-specific candidate variants.
Note that 13172 SNVs on 120 chromosomes yield 13052 distances.

Distance between adjacent SNVs (bp)	N of SNVs	Cumulative total	Cumulative %
1	58	58	0.4
2	62	120	0.9
3	50	170	1.3
4	66	236	1.8
5	66	302	2.3
6	38	340	2.6
7	29	369	2.8
8	33	402	3.1
9	27	429	3.3
10	28	457	3.5
11	25	482	3.7
12	28	510	3.9
13	15	525	4.0
14	17	542	4.2
15	20	562	4.3
16	11	573	4.4
17	10	583	4.5
18	16	599	4.6
19	8	607	4.7
20	18	625	4.8
21	15	640	4.9
22	10	650	5.0
23	5	655	5.0
24	9	664	5.1
25	10	674	5.2
26-1000	683	1357	10.4
1001-10,000	970	2327	17.8
10001-100,000	1696	4023	30.8
100,001-1,000,000	5448	9471	72.6
1,000,001-45,000,000	3581	13052	100.0

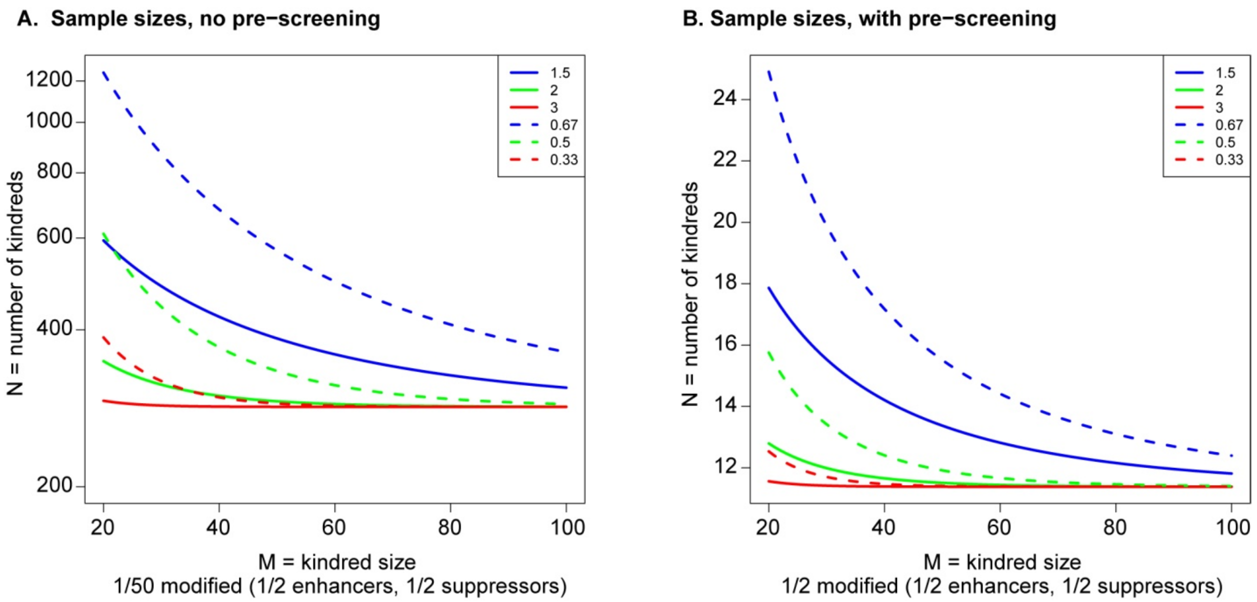

Figure S1 Sample size requirements in a progeny test for various fold effects modifying the expected tumor count, both (A) without prescreening, assuming 1/100 gametes have a modifier with directional effect shown, and (B) with survival-based pre-screening such that ¼ of gametes have directional effect shown. Sample sizes are calculated such that a 5% FDR-controlled list of modified kindreds is non-empty with 95% probability. In all cases the non-modified tumor-count distribution is Negative Binomial, with mean 99.8 tumors and shape parameter 9.8, as estimated from control data. Modifiers are assumed to affect the mean (and thus the variance), but not the shape parameter. (Recall that a Negative Binomial distribution has mean mu and variance mu*(1+mu/shape)). Calculations allow segregation of each mutant modifier within a carrier kindred and use a normal approximation for the distribution of average tumor count. We reckoned that a one-hit mutagenesis library will produce 1/50 gametes carrying some modifier, and that ½ of these may be in a specific direction, and this determined the rates used above. Without pre-screening, the burden of a tumor-count-based progeny test is especially high in terms of the number of kindreds required to be tested.

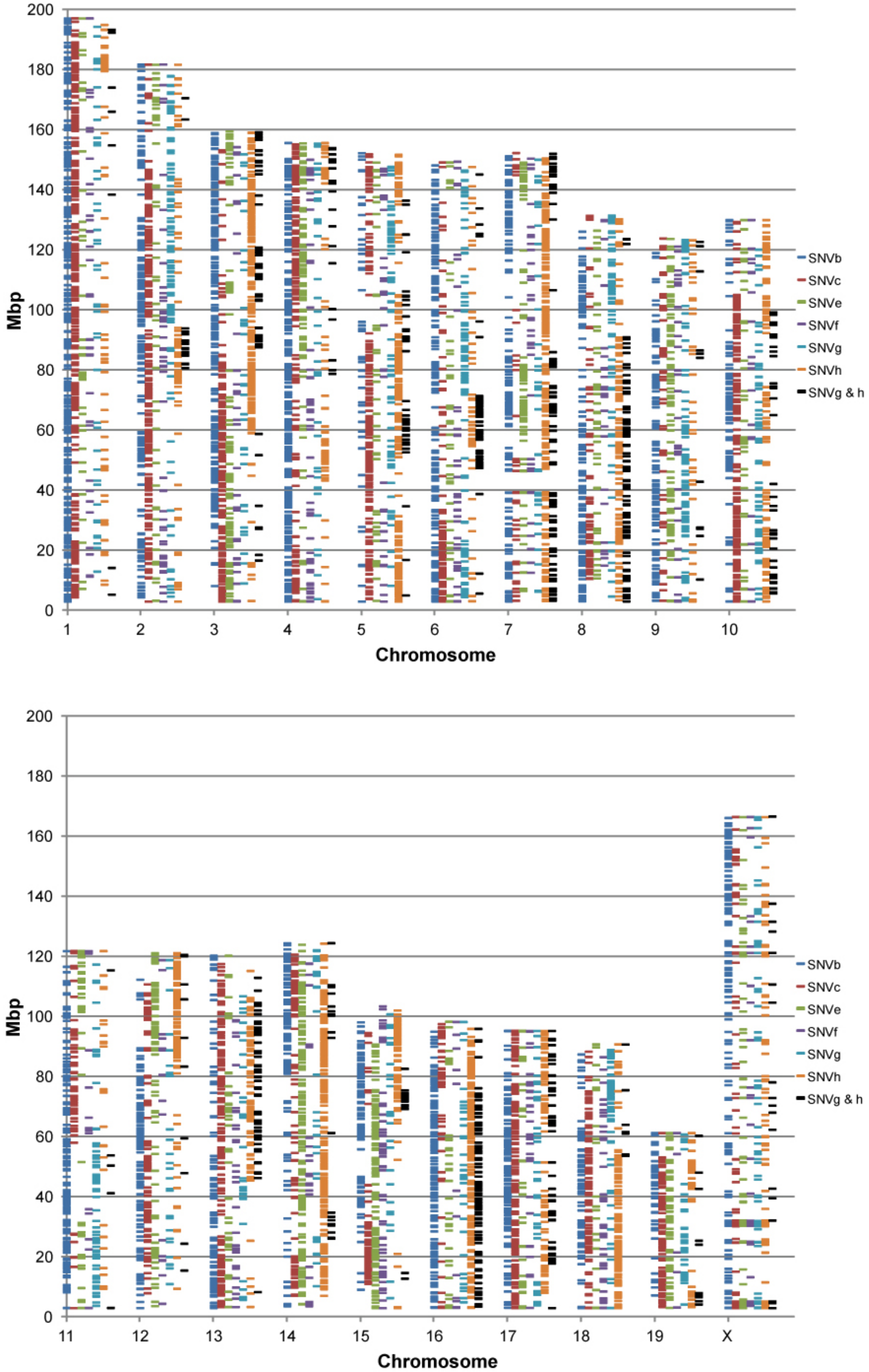

Figure S2 The candidate SNV sites that are line-specific or specific to only lines B6.SNVg and B6.SNVh.

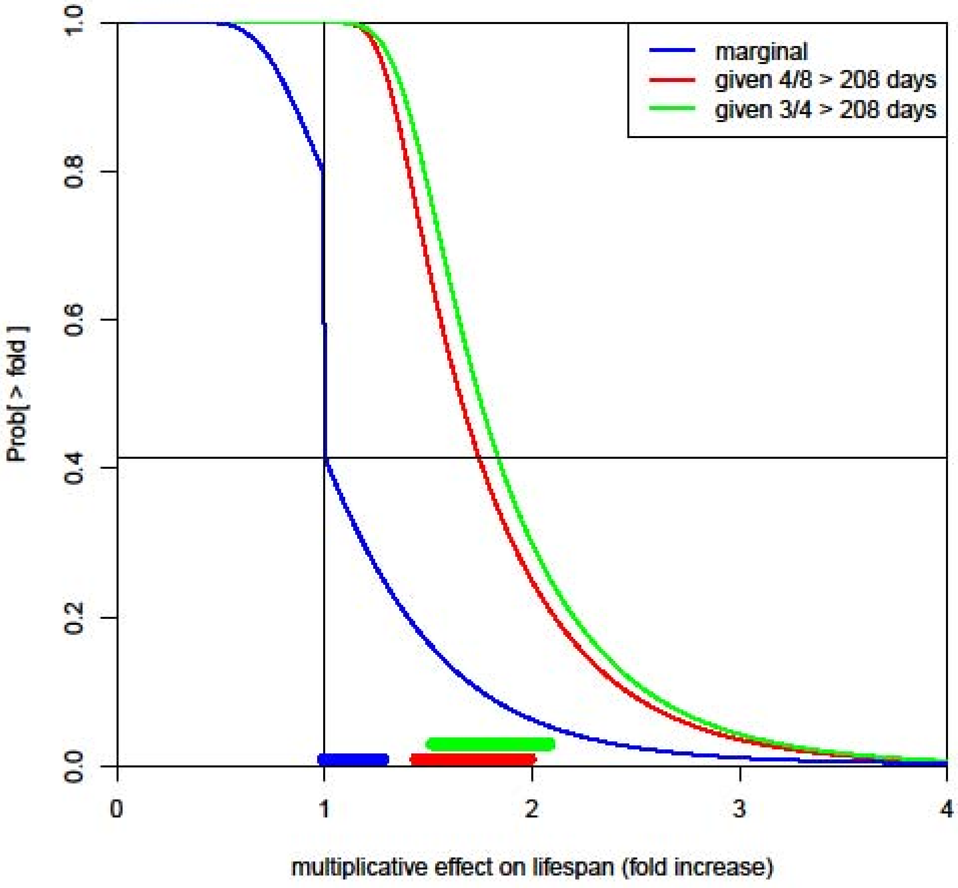

Figure S3 $P(M > f | \text{SEL})$: how selecting on long-lived phenotype enriches for large fold effects. Thick lines show interquartile range of *M* values (as for kindreds 201 and 258)

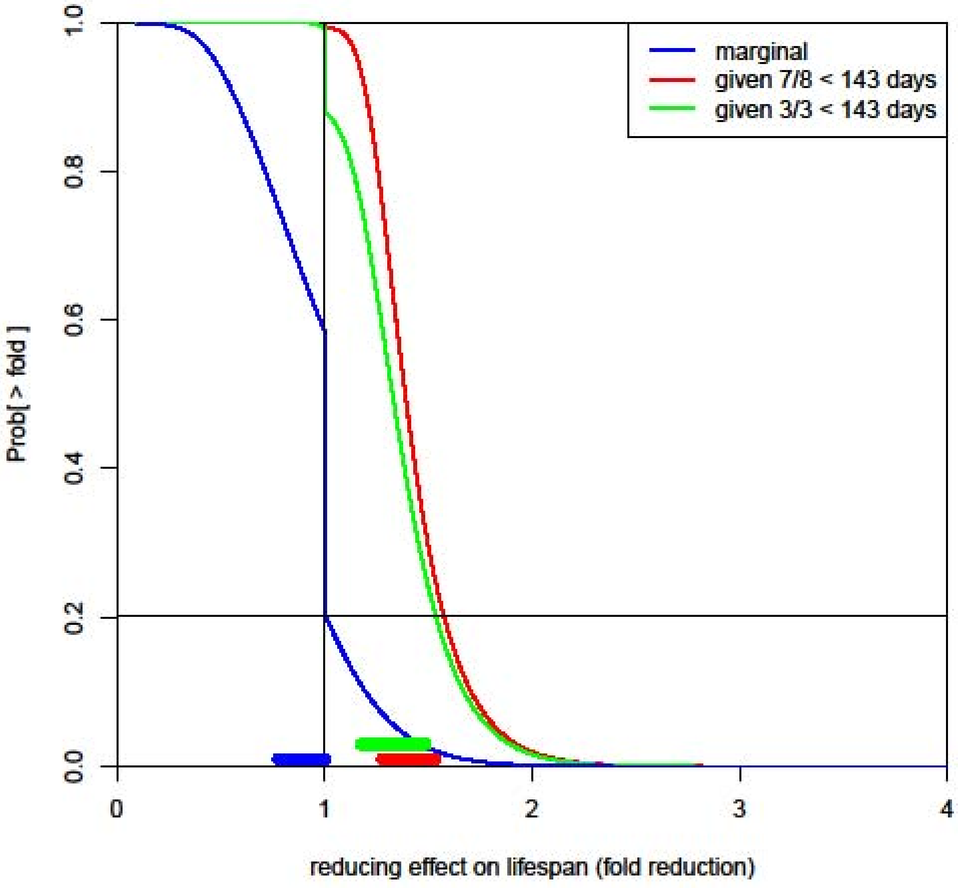

Figure S4 $P(1/M > f|\mathrm{SEL})$: how selecting on short-lived phenotype enriches for small fold effects. Thick lines show interquartile range of *M* values (as for kindreds 333, 415)

File S1

Supporting Data

Illumina sequencing results for lines B6.SNVb, B6.SNVc, B6.SNVe, B6.SNVf, B6.SNVg and B6.SNVh

Paired-end 75bp reads were performed on the Illumina GAIIx platform and mapped to the mm9/NCBIm37 assembly as described in Methods. Major changes in copy number or sequencing efficiency were culled by filtering out signals stronger than 2.5 times the sequencing coverage of the sample.

The data are contained in File S1 which is available for download at http://www.g3journal.org/lookup/suppl/doi:10.1534/g3.114.010595/-/DC1.

File S2

Supporting Methods

Modeling lifespans of mutagenized G1F1 animals

Considerable data were available on the lifespans of animals who are possible carriers of ENU-induced dominant modifiers of *Apc*^{Min}, as summarized in Figure 3 of the main paper. In total n=1525 G1F1 lifespans were measured. Because the sampled population is a mixture of those affected and unaffected by Apc modifiers, the G1F1 lifespan distribution is also informed by n=42 observed lifespans from control F1 animals (unaffected by any modifiers) and data on the directional effect of confirmed modifiers (Table 3, Kwong and Dove, 2009). File S2, available for download, describes in detail a statistical analysis of these three data sources in terms of how they inform the likely lifespan effects of modifiers. Specifically, we assume that a lifespan, X, may be expressed by $X = X_0 M$, where X_0 is the counter-factual average lifespan the animal would have experienced if it carried no mutant modifier, and M is the multiplicative effect of the modifier, assumed to be independent of X_0. The calculation represents the mutagenized G1F1 lifespan distribution as a mixture of three components, corresponding to unaffected animals (M=1), long-lived animals (M>1), and short-lived animals (M<1). Using a novel log-normal discrete-mixing formulation, a maximum likelihood estimate is obtained of the mixture distribution. This informs both the marginal distribution of effects M, and also the conditional distribution of M given kindreds selected on the basis of multiple animals having an extreme long-term or short-term survival phenotype. It finds that a large proportion of mutagenized gametes carry modifiers M not equal to 1, but that without selection the typical size of M is expected to be quite small.

The statistical methods are contained in File S2 which is available for download at http://www.g3journal.org/lookup/suppl/doi:10.1534/g3.114.010595/-/DC1.

File S3

Supporting Methods

Mutagenesis sample-size planning

Consider a mutagenesis study involving *N* gametes, each possibly carrying a mutation that modifies the expected intestinal tumor count in Apc^{Min}-carrying mice. Many mutations may be phenotypically silent, but a fraction π affect the mean tumor count in carriers (with a 1-hit library we expect about 1/50 of gametes to have a modifier, possibly with 1/2 enhancers and 1/2 suppressors of the tumor phenotype). Each gamete may be progeny tested using *M* animals in order to assess its modifier status.

We assume a negative binomial (NB) distribution on tumor counts, with mutations affecting the mean but not the shape, with the baseline parameters estimated from pilot data. Two calculations are considered below. The first asks how large should be *M*, the number of animals in a progeny test of a single gamete, in order to have high power to detect a true modifier. This we call fully testing a gamete. The second question considers how to process data from a series of gametes in order to have high probability that an FDR controlled list of putative modifiers is nonempty. The number of kindreds (gametes) required, *N*, depends also on the rate of occurrence of modifiers in the gamete stream, which may be low in a 1-hit library, or which may be enriched via pre-screening according to a surrogate phenotype (e.g. survival).

The statistical methods are contained in File S3 which is available for download at http://www.g3journal.org/lookup/suppl/doi:10.1534/g3.114.010595/-/DC1.

File S4

Supporting Data

Results of Sequenom validation tests of DNA from 31 C57BL6/6JD-*Apc*^{Min}, 6 B6.SNVg, 1 B6.SNVh, and 41 (C57BL/6JD-*Apc*^{Min} x B6.SNVg)F1 mice. Note that 14 of the samples were run twice (gray-coded animal ID cells). Data from B6 and B6.SNVg sequences are shown in columns D and E, respectively. Unexpected Sequenom results are highlighted in red. For 11 of the positions, the unexpected results in the B6.SNVg mice and/or the F1s are consistent with residual heterozygosity in the B6.SNVg line (see comment column). The unexpected results at 5 positions are consistent with either an error in the canonical B6 sequence or genetic drift in C57BL/6JD-*Apc*^{Min}.

File S4 is available for download at http://www.g3journal.org/lookup/suppl/doi:10.1534/g3.114.010595/-/DC1.
