# Supplementary material for: A Strategy to Identify Dominant Point Mutant Modifiers of a Quantitative Trait
Source: G3 (Bethesda). 2014 Apr 17;4(6):1113–21. doi: 10.1534/g3.114.010595 (PMC4065254; doi:10.1534/g3.114.010595)
Supplement: Supporting Information [file supp_g3.114.010595_TableS1.pdf]

Table S1 Sequencing of the B6-SNV lines.

Line	Raw (Gbp)	Mapped (Gbp)	Coverage (times)	N of all candidate SNVs[a]	N of line-specific candidate SNVs[b]
SNVb	27.40	23.83	7.94	9864	3246
SNVc	20.61	17.21	5.74	9028	2932
SNVe	14.64	12.24	4.08	7094	2051
SNVf	6.05	5.14	1.71	2925	1198
SNVg	28.38	24.25	8.08	6324	1225
SNVh	23.85	20.03	6.68	9559	2520

[a]There were 22911 sites overall: 13172 appeared in only 1 line, 3590 in 2, 2187 in 3, 2212 in 4, 1467 in 5, and 283 in all 6 lines.

[b]In addition, lines B6.SNVg and B6.SNVh, which are known to be related, share 792 candidate variants that are found in none of the other lines.
