# Supplementary material for: A Strategy to Identify Dominant Point Mutant Modifiers of a Quantitative Trait
Source: G3 (Bethesda). 2014 Apr 17;4(6):1113–21. doi: 10.1534/g3.114.010595 (PMC4065254; doi:10.1534/g3.114.010595)
Supplement: Supporting Information [file supp_g3.114.010595_TableS2.pdf]

Table S2 The spectrum of distances between 13172 adjacent line-specific candidate variants.
Note that 13172 SNVs on 120 chromosomes yield 13052 distances.

**Distance between adjacent SNVs (bp)**	**N of SNVs**	**Cumulative total**	**Cumulative %**
1	58	58	0.4
2	62	120	0.9
3	50	170	1.3
4	66	236	1.8
5	66	302	2.3
6	38	340	2.6
7	29	369	2.8
8	33	402	3.1
9	27	429	3.3
10	28	457	3.5
11	25	482	3.7
12	28	510	3.9
13	15	525	4.0
14	17	542	4.2
15	20	562	4.3
16	11	573	4.4
17	10	583	4.5
18	16	599	4.6
19	8	607	4.7
20	18	625	4.8
21	15	640	4.9
22	10	650	5.0
23	5	655	5.0
24	9	664	5.1
25	10	674	5.2
26-1000	683	1357	10.4
1001-10,000	970	2327	17.8
10001-100,000	1696	4023	30.8
100,001-1,000,000	5448	9471	72.6
1,000,001-45,000,000	3581	13052	100.0
