# Supplementary material for: A Strategy to Identify Dominant Point Mutant Modifiers of a Quantitative Trait
Source: G3 (Bethesda). 2014 Apr 17;4(6):1113–21. doi: 10.1534/g3.114.010595 (PMC4065254; doi:10.1534/g3.114.010595)
Supplement: Supporting Information [file supp_g3.114.010595_FigureS1.pdf]

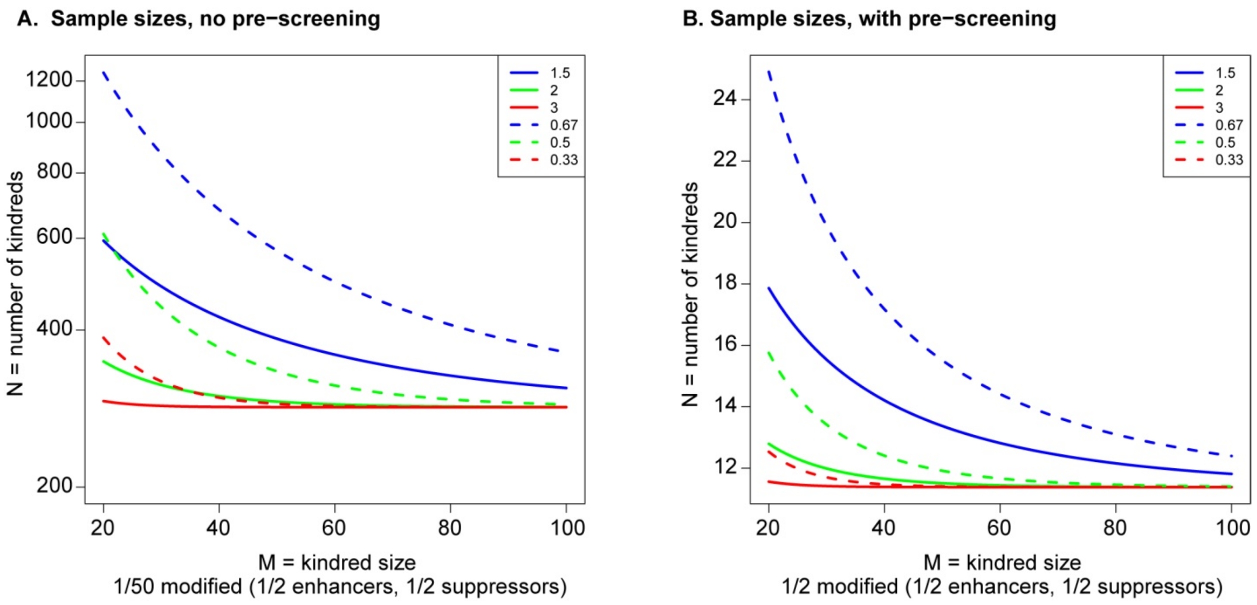

Figure S1 Sample size requirements in a progeny test for various fold effects modifying the expected tumor count, both (A) without prescreening, assuming 1/100 gametes have a modifier with directional effect shown, and (B) with survival-based pre-screening such that ¼ of gametes have directional effect shown. Sample sizes are calculated such that a 5% FDR-controlled list of modified kindreds is non-empty with 95% probability. In all cases the non-modified tumor-count distribution is Negative Binomial, with mean 99.8 tumors and shape parameter 9.8, as estimated from control data. Modifiers are assumed to affect the mean (and thus the variance), but not the shape parameter. (Recall that a Negative Binomial distribution has mean mu and variance mu*(1+mu/shape)). Calculations allow segregation of each mutant modifier within a carrier kindred and use a normal approximation for the distribution of average tumor count. We reckoned that a one-hit mutagenesis library will produce 1/50 gametes carrying some modifier, and that ½ of these may be in a specific direction, and this determined the rates used above. Without pre-screening, the burden of a tumor-count-based progeny test is especially high in terms of the number of kindreds required to be tested.
