# Supplementary material for: A Strategy to Identify Dominant Point Mutant Modifiers of a Quantitative Trait
Source: G3 (Bethesda). 2014 Apr 17;4(6):1113–21. doi: 10.1534/g3.114.010595 (PMC4065254; doi:10.1534/g3.114.010595)
Supplement: Supporting Information [file supp_g3.114.010595_FigureS3.pdf]

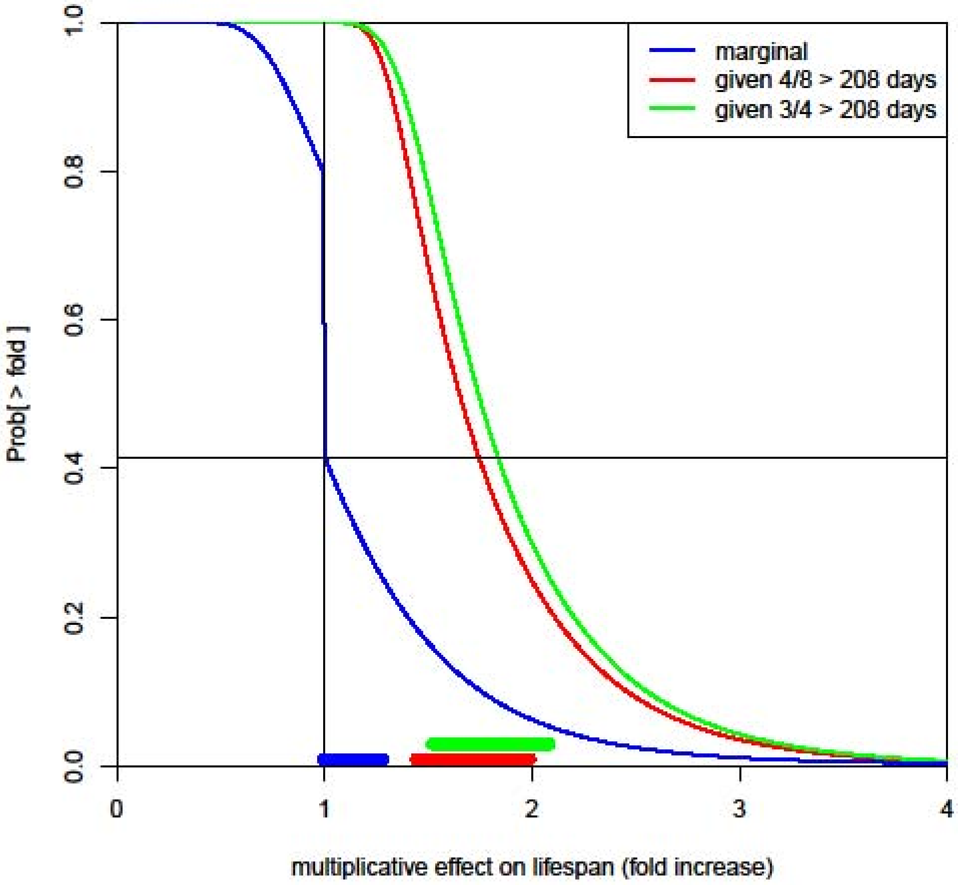

Figure S3 $P(M > f|\text{SEL})$: how selecting on long-lived phenotype enriches for large fold effects. Thick lines show interquartile range of *M* values (as for kindreds 201 and 258)
