# Supplementary material for: A Strategy to Identify Dominant Point Mutant Modifiers of a Quantitative Trait
Source: G3 (Bethesda). 2014 Apr 17;4(6):1113–21. doi: 10.1534/g3.114.010595 (PMC4065254; doi:10.1534/g3.114.010595)
Supplement: Supporting Information [file supp_g3.114.010595_FigureS4.pdf]

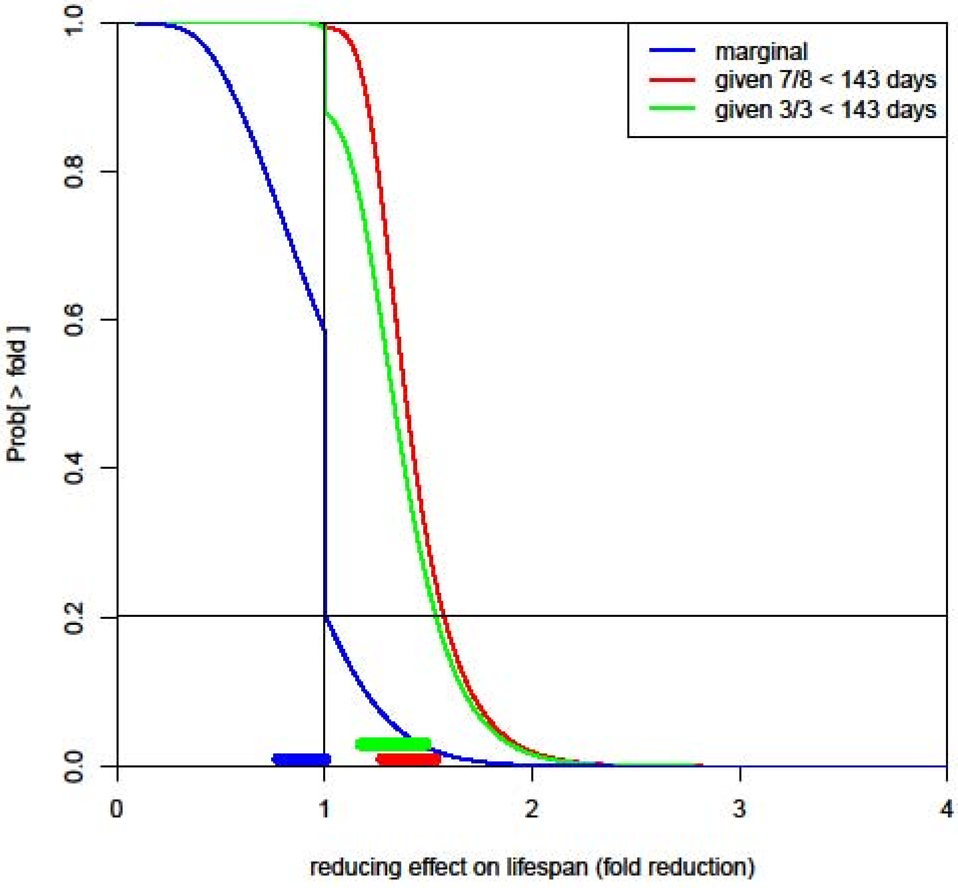

Figure S4 $P(1/M > f|\text{SEL})$: how selecting on short-lived phenotype enriches for small fold effects. Thick lines show interquartile range of *M* values (as for kindreds 333, 415)
