# Supplementary material for: A Strategy to Identify Dominant Point Mutant Modifiers of a Quantitative Trait
Source: G3 (Bethesda). 2014 Apr 17;4(6):1113–21. doi: 10.1534/g3.114.010595 (PMC4065254; doi:10.1534/g3.114.010595)
Supplement: Supporting Information [file supp_g3.114.010595_FileS1.zip › FileS1/READ_ME.pdf]

File S1

Supporting Data

Illumina sequencing results for lines B6.SNVb, B6.SNVc, B6.SNVe, B6.SNVf, B6.SNVg and B6.SNVh

Paired-end 75bp reads were performed on the Illumina GAIIx platform and mapped to the mm9/NCBIm37 assembly as described in Methods. Major changes in copy number or sequencing efficiency were culled by filtering out signals stronger than 2.5 times the sequencing coverage of the sample.
