# Supplementary material for: A Strategy to Identify Dominant Point Mutant Modifiers of a Quantitative Trait
Source: G3 (Bethesda). 2014 Apr 17;4(6):1113–21. doi: 10.1534/g3.114.010595 (PMC4065254; doi:10.1534/g3.114.010595)

Supporting File S2: Modeling lifespans of mutagenized G1F1 animals

1 Mixture model

Fully $n = 1525$ G1F1 animals were followed for the lifespan phenotype: X_i, for animal i. Some fraction π_0 of lifespans were unaffected by any mutagenesis-induced modifiers, and their lifespan density, $f_0(x)$, say, is taken to be a log-normal, with parameters μ_0 and σ_0^2. Some other fraction π_{LL} carried a mutant modifier that induced a *long-life* phenotye, while yet a third fraction π_{SL} carried a mutant modifier that induced a *short-life* phenotype. Denoting these component densities by $f_{LL}(x)$ and $f_{SL}(x)$ respectively, we view the density of X_i in the G1F1 population as a mixture:

$$f_{\text{mix}}(x) = \pi_0 f_0(x) + \pi_{LL} f_{LL}(x) + \pi_{SL} f_{SL}(x).$$

In preliminary calculations we treated all three components as log-normal, and while this produced a well-fitting model (fit by the `R` package `mclust`), the model was poorly adapted to the primary inference task concerning expected modifier effects. The fit suggested a large fraction of modifiers whose effect on the phenotype must be relatively small. Yet it was difficult within the log-normal mixture formulation to characterize either the marginal distribution of modifier effects or the conditional distribution of effects given selection of animals with extreme phenotypes.

We gain ground by adapting a useful idea from human survival analysis, specifically the *accelerated failure time* (AFT) model. Animal i's lifetime X_i, in case it is experiencing a mutation-modified phenotype, is assumed to be $X_i = X_{0,i} M_i$, where $X_{0,i} \sim f_0$ is a counter-factual lifespan the animal would have had in the absence of the modifier, and M_i is the fold-effect that the modifier has on animal i's lifespan. Indeed, for the LL component, $M_i > 1$ and for the SL component $0 < M_i < 1$. We make progress by further structuring the law of M_i as a constrained log-normal factor:

$$\log M_i \mid \text{LL} \;\sim\; \text{Normal}\left(0, \sigma_{LL}^2\right) 1[M_i > 1]$$

and

$$\log M_i \mid \text{SL} \;\sim\; \text{Normal}\left(0, \sigma_{SL}^2\right) 1[0 < M_i < 1].$$

Parameters σ_{LL} and σ_{SL} characterize the expected magnitude of modifier effects. With these assumptions, it is possible to integrate the latent M_i and get a closed formula for the component densities $f_{LL}(x)$ and $f_{SL}(x)$. For convenience, we report these on the log-scale, for lifetimes $Y_i = \log X_i$. With ϕ and Φ the standard normal density and cumulative distribution functions, respectively, we derive the following:

$$f_{LL}(y) = \frac{2}{\sqrt{\sigma_0^2 + \sigma_{LL}^2}} \phi\left[\frac{y - \mu_0}{\sqrt{\sigma_0^2 + \sigma_{LL}^2}}\right] \Phi\left[\frac{y - \mu_0}{\sigma_0^2 \sqrt{\frac{1}{\sigma_0^2} + \frac{1}{\sigma_{LL}^2}}}\right]$$

and

$$f_{SL}(y) = \frac{2}{\sqrt{\sigma_0^2 + \sigma_{SL}^2}} \phi\left[\frac{y - \mu_0}{\sqrt{\sigma_0^2 + \sigma_{SL}^2}}\right] \Phi\left[\frac{\mu_0 - y}{\sigma_0^2 \sqrt{\frac{1}{\sigma_0^2} + \frac{1}{\sigma_{SL}^2}}}\right].$$

The first factor in each density is the marginal density for Y_i in the no-constraint model; the second factor is the posterior probability that the constraint holds. This interesting relationship appears in various constructions of mixture components defined by constraints on random effects, and is the subject of a working manuscript by MAN.

2 Integrating three data sets

We used maximum likelihood estimation to fit the mixture model in Section 1, and to inform the estimation as much as possible we integrated three data sets:

- $n = 1525$ G1F1 lifetimes
- $n = 42$ F1 lifetimes
- $n = 31$ modifier direction calls (Table 3, Kwong and Dove, 2009)

The $n = 1525$ G1F1 lifetimes arise from the full mixture; we don't know which class any one comes from. Their contribution to the log-likelihood is:

$$\sum_{i \in \mathrm{G1F1}} \log f_{\mathrm{mix}}(x_i).$$

The $n = 42$ F1 lifetimes ought to follow the unaffected distribution f_0; including these helps to anchor the mixture. Their contribution to the log-likelihood is

$$\sum_{i \in \mathrm{F1}} \log f_0(x_i).$$

Further, the rate at which modifiers might enhance or suppress the lifetime phenotype is informed by extensive data on the directional effects of previously identified Min modifiers. For $n = 31$ unequivacal cases in Table 3 of Kwong and Dove (2009), we know the directional effect on tumor multiplicity (one case is equivocal, affecting multiplicity differently in the small intestine and colon, and we omit that case). Of the remaining $n = 31$, $n_{SL} = 21$ increase tumor multiplicity, and thus would be expected to reduce lifespan, and $n_{LL} = 10$ decrease multiplicity and thus would be expected to increase lifespan. These numbers inform the mixing parameters π_{LL} and π_{SL}. We treat them as Bernoulli trials from the conditional distribution of the class indicators given that the class is either LL or SL, and thus their log-likelihood contribution is:

$$n_{SL} \log \pi_{SL} + n_{LL} \log \pi_{LL} - (n_{SL} + n_{LL}) \log(\pi_{SL} + \pi_{LL})$$

We use the R function `nlminb` to optimize the log-likelihood, which combines all three data sets and parameters: μ_0, σ_0, σ_{LL}, σ_{SL}, π_0, π_{LL}, π_{SL}. Maximum likelihood estimates are shown in Table 1. The model fit is shown in Figure 3 (main paper). Note that by segregation in the G1F1, the rate at which G1F1 kindred parents carry modifiers is $2\pi_{LL}$ (for long-lived effects) and $2\pi_{SL}$ for short-lived effects.

Table 1: MLEs

$\hat{\mu}_0$	$\hat{\sigma}_0$	$\hat{\sigma}_{LL}$	$\hat{\sigma}_{SL}$	$\hat{\pi}_0$	$\hat{\pi}_{LL}$	$\hat{\pi}_{SL}$
5.046	0.168	0.477	0.261	0.691	0.207	0.102

We experimented with elaborations of the model, allowing non-zero means on the unconstrained distribution of $\log M_i$, but BIC statistics lead us to retain this basic model described above.

3 Enhancing modifier effects by selecting extreme phenotypes

The G1F1 population is comprised of multiple kindreds. The mixture above concerns animal-level data, but we recognize that the kindred is the unit that has inherited (or not) a mutant modifier of effect M. In kindreds showing multiple animals having an extreme phenotype, there is an enrichment for the likely modifier effect. This is demonstrated in Figure S3, using two selection rules (corresponding to kindreds 258 and 201 with multiple long-lived animials). The calculation rests on the AFT model, and considers:

$$P(M > f|\text{SEL}) \tag{1}$$

for various fold changes f. For kindred 258, SEL is the event that at least 4 of 8 animals are long-lived. For kindred 201, SEL is the event that at least 3 of 4 animals are long-lived. The probability uses the fact that multiple lifetimes within a given kindred share the same multiplier M, so $X_i = X_{0,i}M$ for all animals i in the kindred. The conditional exceedance probabilties are then computed using elements of the fitted mixture (see equation 2 below). We see from Figure S3 that selection based upon multiple long-lived sibs greatly enhances the magnitude of the underlying fold effect M, whose unselected distribution is shown in blue. The interquartile range (IQR) (thick lines) brackets unity (no effect) and allows only modest effects, even though the fitted model estimates that 41% of the modifiers extend lifespan (twice the animal rate). I.e. there are many very small effects. On the other hand, the enriched population has a greatly shifted distribution, and IQRs containing 2 fold. Figure S4 shows the comparable plot for short-lived modifiers; here about 20% of the kindreds carry such a modifier, though the modifier effects are quite small and the selection examples do not enrich for very large effect magnitudes.

Here we give a formula for the enrichment effect on the long-lived side (1); the opposite side is comparable. We consider specifically the selection based upon a kindred of n siblings, at least k of whom have lifespan exceeding c.

$$P\left[M > f|\text{SEL}(n,k,c)\right] = 2\pi_{LL}K(n,k,c)\int_{\log f}^{\infty} \psi(n,k,c,u)\frac{1}{\sigma_{LL}}\phi\left(\frac{u}{\sigma_{LL}}\right)du \tag{2}$$

where ψ accounts for the within-kindred selection multiplicities

$$\psi(n,k,c,u) = \sum_{j=k}^{n}\binom{n}{j}\beta^j(1-\beta)^{n-j}$$

and further, β is the chance that a single animal exceeds the selection threshold, conditional on the fold $\exp(u)$

$$\beta = 1 - \Phi\left[\frac{\log(c) - u - \mu_0}{\sigma_0}\right],$$

and finally $K(n,k,c)$ is a normalizing constant equaling the inverse of the marginal selection probability, which does not involve the fold f.

Figure S3: $P(M > f|\text{SEL})$: how selecting on long-lived phenotype enriches for large fold effects. Thick lines show interquartile range of M values (as for kindreds 201 and 258)

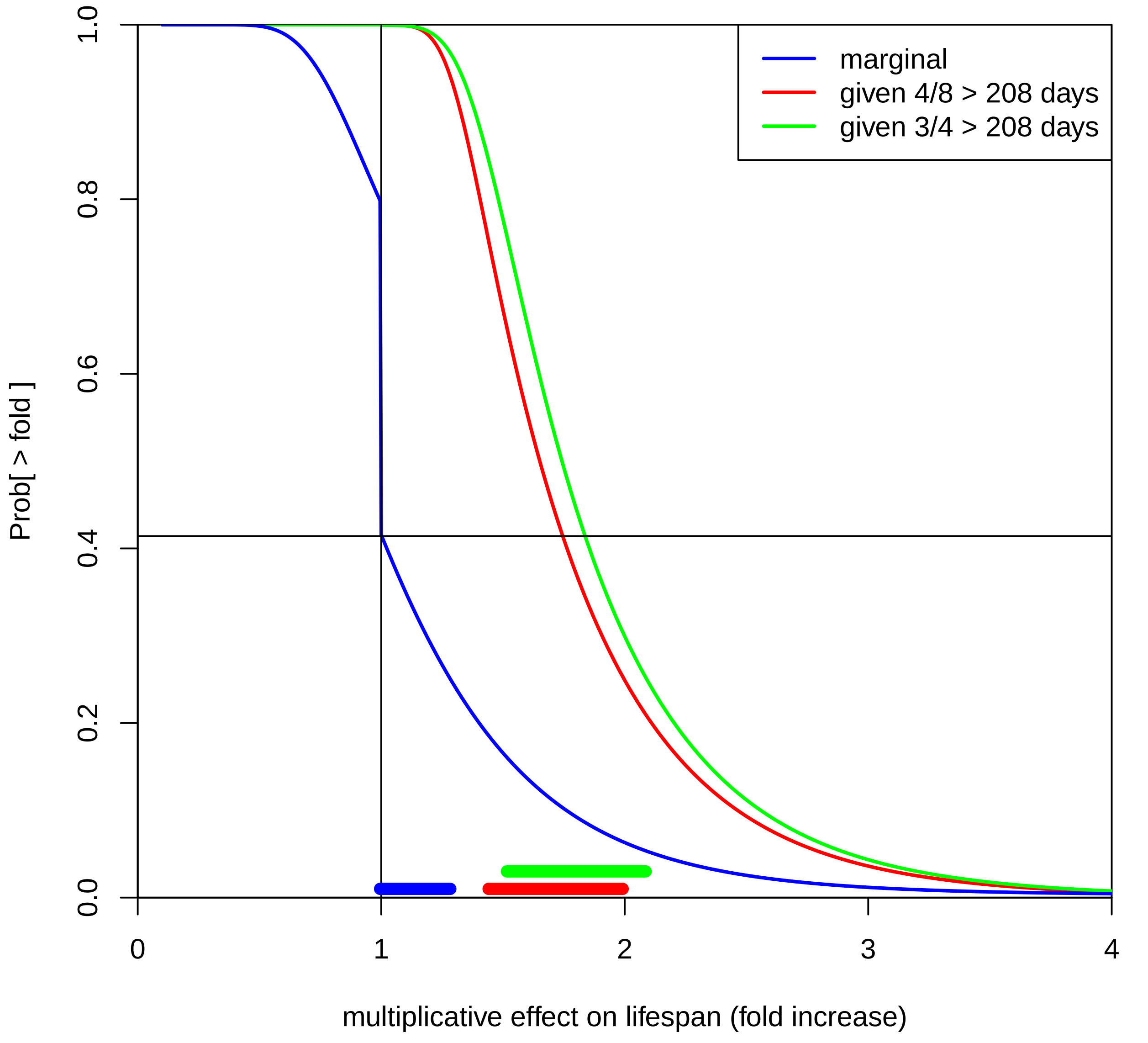

Figure S4: $P(1/M > f|\text{SEL})$: how selecting on short–lived phenotype enriches for small fold effects. Thick lines show interquartile range of M values (as for kindreds 333, 415)

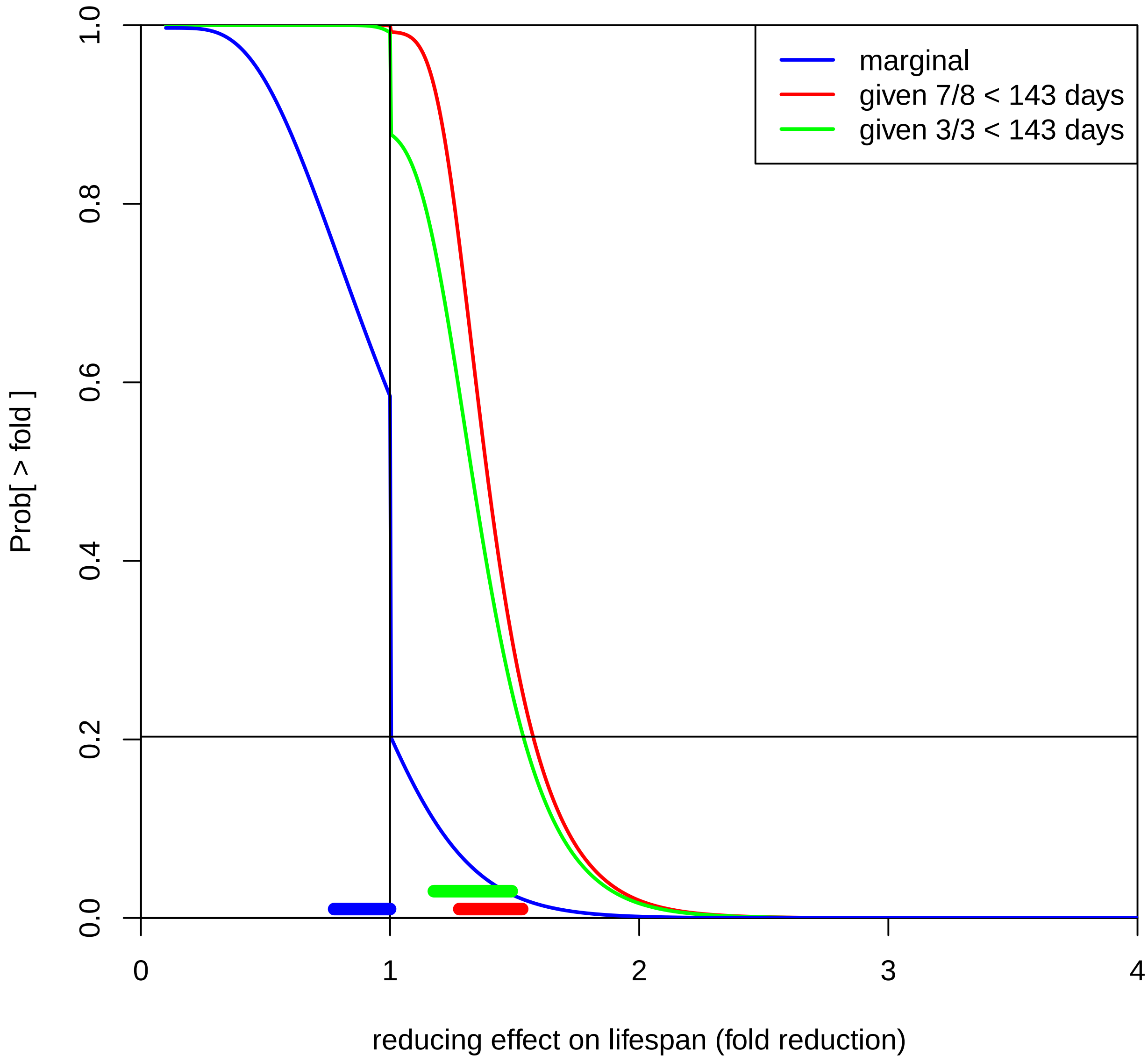

Supplement: Supporting Information [file supp_g3.114.010595_FileS2.zip › FileS2/FileS2.pdf]
