# Supplementary material for: A Strategy to Identify Dominant Point Mutant Modifiers of a Quantitative Trait
Source: G3 (Bethesda). 2014 Apr 17;4(6):1113–21. doi: 10.1534/g3.114.010595 (PMC4065254; doi:10.1534/g3.114.010595)
Supplement: Supporting Information [file supp_g3.114.010595_FileS2.zip › FileS2/READ_ME.pdf]

File S2

Supporting Methods

Modeling lifespans of mutagenized G1F1 animals

Considerable data were available on the lifespans of animals who are possible carriers of ENU-induced dominant modifiers of *Apc*^{Min}, as summarized in Figure 3 of the main paper. In total n=1525 G1F1 lifespans were measured. Because the sampled population is a mixture of those affected and unaffected by Apc modifiers, the G1F1 lifespan distribution is also informed by n=42 observed lifespans from control F1 animals (unaffected by any modifiers) and data on the directional effect of confirmed modifiers (Table 3, Kwong and Dove, 2009). File S2, available for download, describes in detail a statistical analysis of these three data sources in terms of how they inform the likely lifespan effects of modifiers. Specifically, we assume that a lifespan, X, may be expressed by $X = X_0 M$, where X_0 is the counter-factual average lifespan the animal would have experienced if it carried no mutant modifier, and M is the multiplicative effect of the modifier, assumed to be independent of X_0. The calculation represents the mutagenized G1F1 lifespan distribution as a mixture of three components, corresponding to unaffected animals (M=1), long-lived animals (M>1), and short-lived animals (M<1). Using a novel log-normal discrete-mixing formulation, a maximum likelihood estimate is obtained of the mixture distribution. This informs both the marginal distribution of effects M, and also the conditional distribution of M given kindreds selected on the basis of multiple animals having an extreme long-term or short-term survival phenotype. It finds that a large proportion of mutagenized gametes carry modifiers M not equal to 1, but that without selection the typical size of M is expected to be quite small.
