# Supplementary material for: A Strategy to Identify Dominant Point Mutant Modifiers of a Quantitative Trait
Source: G3 (Bethesda). 2014 Apr 17;4(6):1113–21. doi: 10.1534/g3.114.010595 (PMC4065254; doi:10.1534/g3.114.010595)
Supplement: Supporting Information [file supp_g3.114.010595_FileS3.zip › FileS3/FileS3.pdf]

Supporting File S3: Mutagenesis sample-size planning

1 Summary

Consider a mutagenesis study involving N gametes, each possibly carrying a mutation that modifies the expected intestinal tumor count in Apc-carrying mice. Many mutations may be phenotypically silent, but a fraction π affect the mean tumor count in carriers; with a 1-hit library we expect about 1/50 of gametes to have a modifier. Each gamete may be progeny tested using M animals in order to assess its modifier status.

We assume a negative binomial (NB) distribution on tumor counts, with mutations affecting the mean but not the shape, with the baseline parameters estimated from pilot data. Two calculations are considered below. The first asks how large should be M, the number of animals in a progeny test of a single gamete, in order to have high power to detect a true modifier. This we call fully testing a gamete. The second question considers how to process data from a series of gametes in order to have high probability that an FDR controlled list of putative modifiers is nonempty. The number of kindreds (gametes) required, N, depends also on the rate of occurence of modifiers in the gamete stream, which may be low in a 1-hit library, or which may be enriched via pre-screening according to a surrogate phenotype (e.g. survival).

2 Calculation

2.1 Model

Let $i = 1, 2, \ldots, N$ index kindreds, $j = 1, 2, \ldots M$ index progeny test animals within each kindred, and $X_{i,j}$ denote the intestinal tumor count in animal (i, j). The presence of a mutant modifier of Apc Min (e.g., enhancer) is indicated by the Bernoulli trial Z_i, with $E(Z_i) = \pi$. In the absence of an active modifier

$$X_{i,j}|Z_i = 0 \sim \text{NB}(\text{mean} = \ \mu_0, \text{ shape} = \sigma)$$

Note, the variance of NB is $V_0 = \mu_0(1 + \mu_0/\sigma)$, and that increasing σ drives convergence to the Poisson distribution.

The effect of an enhancer is to boost the expected tumor count, and we assume, for an effect $\phi > 1$,

$$X_{i,j}|U_{i,j} = 1 \sim \text{NB}(\text{mean} = \ \mu_1 = \phi\mu_0, \text{ shape} = \sigma).$$

Here $U_{i,j}$ is the Bernoulli trial indicating that the modifier has segregated into animal j. It has mean 1/2 if $Z_i = 1$ and mean 0 if $Z_i = 0$. Let $V_1 = \mu_1(1 + \mu_1/\alpha)$ denote the variance of counts when $U_{i,j} = 1$.

2.2 Kindred statistic

On kindred i, the average tumor count $\bar{X}_{i,\cdot}$ is approximately normally distributed for sufficiently large M, so the standardized count

$$Y_i = \frac{\sqrt{M}(\bar{X}_{i,\cdot} - \mu_0)}{\sqrt{V_0}}$$

is approximately standard normal if $Z_i = 0$. If $Z_i = 1$, the mean and variance are affected by the modifier. We compute

$$a = E(Y_i|Z_i = 1) = \sqrt{M}\frac{\mu_0}{\sqrt{V_0}}\frac{(\phi - 1)}{2}$$

and

$$b^2 = \mathrm{var}(Y_i|Z_i = 1) = \frac{1}{2V_0}\left(V_0 + V_1 + \frac{\mu_0^2(1+\phi^2)}{2}\right).$$

For fully testing (question 1), we compute power from these two normal distributions (Figure 5, main), using a 5% significance level and various multiplicative effects ϕ. For processing the series of gametes, the kindred statistic is a mixture of a standard normal (with probability $1 - \pi$) and a shifted and rescaled normal (with probability π). Note that the kindred size M and the effect size ϕ collaborate to boost the standardized effect, which is also reduced the larger is the variance V_0. M does not affect the conditional variance of Y_i; it is curious that the mean shift de-standardizes this variance. This owes to the negative binomial structure involving a mean-variance relationship. Simpler models in which the modifier affects only the mean observation but not its variance would also have not affected the standardized variance.

Next we consider sample-size calculations when testing a series of gametes. Evidence for the presence of a mutant modifier in one kindred i is contained in the value of Y_i, with larger values corresponding to greater evidence. From the mixture structure, and with $\bar{\Phi}(x) = P(Y_i > x|Z_i = 0)$ denoting the standard normal survival function, we know that marginally, for any x:

$$P(Y_i > x) = (1 - \pi)\bar{\Phi}(x) + \pi\bar{\Phi}\left(\frac{x - a}{b}\right).$$

A procedure defined by threshold c_+ is controlled at FDR ψ if $P(Z_i = 0|Y_i > c_+) = \psi$ (or, at least bounded). So, given $\mu_0, \sigma, \pi, \phi, M, \psi$, we can compute the critical threshold c_+ by solving the equation:

$$\psi = \frac{P(Y_i > c_+|Z_i = 0)(1 - \pi)}{P(Y_i > c_+)}.$$

With this threshold in hand, we can ask about operating characteristics, as we would like to have $\theta(M) = P(Y_i > c_+)$ sufficiently large that an experiment involving N kindreds will have a high probability (at least $1 - \beta$, e.g. $\beta = .05$) to have at least one kindred on the FDR-controlled list. The number of kindreds on this list is Binomial$[N, \theta(M)]$, or approximately Poisson with mean $N\theta$. The chance of the list being non-empty is $1 - \exp[N\theta(M)]$, and so the required minimal N is given by:

$$N = \frac{\log(1/\beta)}{\theta(M)}.$$
