# Supplementary material for: A Strategy to Identify Dominant Point Mutant Modifiers of a Quantitative Trait
Source: G3 (Bethesda). 2014 Apr 17;4(6):1113–21. doi: 10.1534/g3.114.010595 (PMC4065254; doi:10.1534/g3.114.010595)
Supplement: Supporting Information [file supp_g3.114.010595_FileS3.zip › FileS3/READ_ME.pdf]

File S3

Supporting Methods

Mutagenesis sample-size planning

Consider a mutagenesis study involving *N* gametes, each possibly carrying a mutation that modifies the expected intestinal tumor count in Apc^{Min}-carrying mice. Many mutations may be phenotypically silent, but a fraction π affect the mean tumor count in carriers (with a 1-hit library we expect about 1/50 of gametes to have a modifier, possibly with 1/2 enhancers and 1/2 suppressors of the tumor phenotype). Each gamete may be progeny tested using *M* animals in order to assess its modifier status.

We assume a negative binomial (NB) distribution on tumor counts, with mutations affecting the mean but not the shape, with the baseline parameters estimated from pilot data. Two calculations are considered below. The first asks how large should be *M*, the number of animals in a progeny test of a single gamete, in order to have high power to detect a true modifier. This we call fully testing a gamete. The second question considers how to process data from a series of gametes in order to have high probability that an FDR controlled list of putative modifiers is nonempty. The number of kindreds (gametes) required, *N*, depends also on the rate of occurrence of modifiers in the gamete stream, which may be low in a 1-hit library, or which may be enriched via pre-screening according to a surrogate phenotype (e.g. survival).
