# Supplementary material for: A Strategy to Identify Dominant Point Mutant Modifiers of a Quantitative Trait
Source: G3 (Bethesda). 2014 Apr 17;4(6):1113–21. doi: 10.1534/g3.114.010595 (PMC4065254; doi:10.1534/g3.114.010595)
Supplement: Supporting Information [file supp_g3.114.010595_FileS4.zip › FileS4/READ_ME.pdf]

File S4

Supporting Data

Results of Sequenom validation tests of DNA from 31 C57BL6/6JD-*Apc*^{Min}, 6 B6.SNVg, 1 B6.SNVh, and 41 (C57BL/6JD-*Apc*^{Min} x B6.SNVg)F1 mice. Note that 14 of the samples were run twice (gray-coded animal ID cells). Data from B6 and B6.SNVg sequences are shown in columns D and E, respectively. Unexpected Sequenom results are highlighted in red. For 11 of the positions, the unexpected results in the B6.SNVg mice and/or the F1s are consistent with residual heterozygosity in the B6.SNVg line (see comment column). The unexpected results at 5 positions are consistent with either an error in the canonical B6 sequence or genetic drift in C57BL/6JD-*Apc*^{Min}.
